# TAC-seq: targeted DNA and RNA sequencing for precise biomarker molecule counting

**DOI:** 10.1038/s41525-018-0072-5

**Published:** 2018-12-18

**Authors:** Hindrek Teder, Mariann Koel, Priit Paluoja, Tatjana Jatsenko, Kadri Rekker, Triin Laisk-Podar, Viktorija Kukuškina, Agne Velthut-Meikas, Olga Fjodorova, Maire Peters, Juha Kere, Andres Salumets, Priit Palta, Kaarel Krjutškov

**Affiliations:** 1 Competence Centre on Health Technologies, Tartu, Estonia; 2 Institute of Biomedicine and Translational Medicine, University of Tartu, Tartu, Estonia; 3 Institute of Molecular and Cell Biology, University of Tartu, Tartu, Estonia; 4 Institute of Computer Science, University of Tartu, Tartu, Estonia; 5 Institute of Clinical Medicine, Department of Obstetrics and Gynaecology, University of Tartu, Tartu, Estonia; 6 Estonian Genome Center, University of Tartu, Tartu, Estonia; 7 Department of Chemistry and Biotechnology, School of Science, Tallinn University of Technology, Tallinn, Estonia; 8 Department of Biosciences and Nutrition, Karolinska Institutet, Huddinge, Sweden; 9 Research Program of Molecular Neurology, Research Programs Unit, University of Helsinki, and Folkhälsan Institute of Genetics, Helsinki, Finland; 10 School of Basic and Medical Biosciences, Guy’s Hospital, King’s College London, London, UK; 11 Institute of Biomedicine and Translational Medicine, Department of Biomedicine, University of Tartu, Tartu, Estonia; 12 Department of Obstetrics and Gynecology, University of Helsinki and Helsinki University Hospital, Helsinki, Finland; 13 Institute for Molecular Medicine Finland, University of Helsinki, Helsinki, Finland

## Abstract

Targeted next-generation sequencing (NGS) methods have become essential in medical research and diagnostics. In addition to NGS sensitivity and high-throughput capacity, precise biomolecule counting based on unique molecular identifier (UMI) has potential to increase biomolecule detection accuracy. Although UMIs are widely used in basic research its introduction to clinical assays is still in progress. Here, we present a robust and cost-effective TAC-seq (Targeted Allele Counting by sequencing) method that uses UMIs to estimate the original molecule counts of mRNAs, microRNAs, and cell-free DNA. We applied TAC-seq in three different clinical applications and compared the results with standard NGS. RNA samples extracted from human endometrial biopsies were analyzed using previously described 57 mRNA-based receptivity biomarkers and 49 selected microRNAs at different expression levels. Cell-free DNA aneuploidy testing was based on cell line (47,XX, +21) genomic DNA. TAC-seq mRNA profiling showed identical clustering results to transcriptome RNA sequencing, and microRNA detection demonstrated significant reduction in amplification bias, allowing to determine minor expression changes between different samples that remained undetermined by standard NGS. The mimicking experiment for cell-free DNA fetal aneuploidy analysis showed that TAC-seq can be applied to count highly fragmented DNA, detecting significant (*p* = 7.6 × 10^−4^) excess of chromosome 21 molecules at 10% fetal fraction level. Based on three proof-of-principle applications we demonstrate that TAC-seq is an accurate and highly potential biomarker profiling method for advanced medical research and diagnostics.

## Biomarkers: Sequencing technique offers accurate RNA or DNA count

A new method for counting individual RNA or DNA molecules could help with biomarker analyses across a range of robust clinical applications. A team led by Kaarel Krjutškov from the Competence Center on Health Technologies in Tartu, Estonia, developed a system called TAC-seq (Targeted Allele Counting by sequencing) that tags RNA transcripts or DNA molecules with strings of random nucleotides, called unique molecular identifiers, to accurately quantify their numbers despite biases introduced by amplification ahead of sequencing. As a proof of principle, the researchers tested the ability of TAC-seq to detect biomarkers important for reproductive medicine. In endometrial biopsies, TAC-seq offered an accurate profile on levels of messenger RNAs and microRNAs linked to a woman’s chance of reproductive success. Proof of principle Down syndrome trisomy detection was carry out using simulated cell-free DNA testing, mimicking non-invasive prenatal genetic testing (NIPT).

## Introduction

Physiological and pathophysiological disease conditions can be often characterized by the precise quantification of specific nucleic acid biomarkers. There are several methods available enabling detection of RNA- or DNA-based biomarkers but recently, next-generation sequencing (NGS) has become one of the favorite approaches because of its high sensitivity, high-throughput, and flexibility. However, despite of its advantages, the relatively high cost of NGS limits its wider application in healthcare. In addition, common NGS assays consist of multiple laboratory steps that often introduce technical biases limiting accurate quantification and, therefore, hinder the robust and clinically valid detection of biomarkers.^1–3^ Although quantitative PCR and digital PCR provide simple and cost-effective alternatives to NGS for quantitative biomarker determination, the multiplexing capacity of these approaches is limited compared to highly parallel NGS-based methods.

To overcome the challenges of precise target quantification on extended scale, NGS sensitivity together with high specificity of ligation-PCR have been compiled into common methods and assays. For example, NGS- and ligation-based TempO-Seq^4^ (Templated Oligo assay with Sequencing readout) and MLPA-seq^5^ are advancement of the well-known MLPA^6^ (Multiplex Ligation-dependent Probe Amplification). Both methods overcome original MLPA multiplexing and detection limitations, and enable to apply sensitivity of NGS and analyze up to 20,000 RNA and 200 genomic DNA (gDNA) targets, respectively. Similar approaches are also RASL-seq^7^ for targeted multiplex messenger RNA (mRNA) analysis and another NGS and targeted ligation-PCR-based method DANSR (digital analysis of selected regions) for cell-free DNA (cfDNA) detection in non-invasive prenatal genetic testing (NIPT). The authors of the latter method report that 384 loci per chromosome 18 and chromosome 21 (altogether 768 loci in a single-tube reaction) are sufficient for aneuploidy discrimination, which makes it significantly less complex assay than so far widely used low-coverage whole-genome sequencing for NIPT.^8^ However, the strategy where ligation-PCR is combined with NGS ensures high level of multiplexing but suffers from the random ligation at low target nucleic acid levels, and on the polymerase-induced errors in both PCR and sequencing steps.^9–11^ As an outcome, above highlighted methods overestimate the original number of studied molecules and are biased by PCR uneven amplification.

To overcome amplification bias in NGS and to maximize nucleic acid detection sensitivity, unique molecular identifiers (UMIs, known also as molecular indexes, unique identifiers or molecular barcodes) are applied. UMI is a string of random nucleotides used in library preparation that was recently introduced to genomic DNA analysis^12^ and also in single-cell transcriptome analysis.^13^ One specific motif out of a large pool of random sequences is incorporated into the original target molecule through oligonucleotides used in library preparation prior to amplification. Later, grouping by identical UMI clones eliminates PCR duplicates and detects the original number of biomolecules. So far, UMIs are widely used in research applications where relatively high PCR amplification is required, such as single-cell analysis and tumor mutation identification.^13–17^ Similarly to above-mentioned applications, UMIs can also be used in targeted ligation-PCR NGS assays to enable absolute quantification of studied biomarker molecules. Taking the previous into account, we developed a ligation- and NGS-based method, TAC-seq, targeted allele counting by sequencing method for original molecule counting of plasma cfDNA and RNA-based biomarkers.

## Results

### TAC-seq principle and assay design

**Fig. 1:**
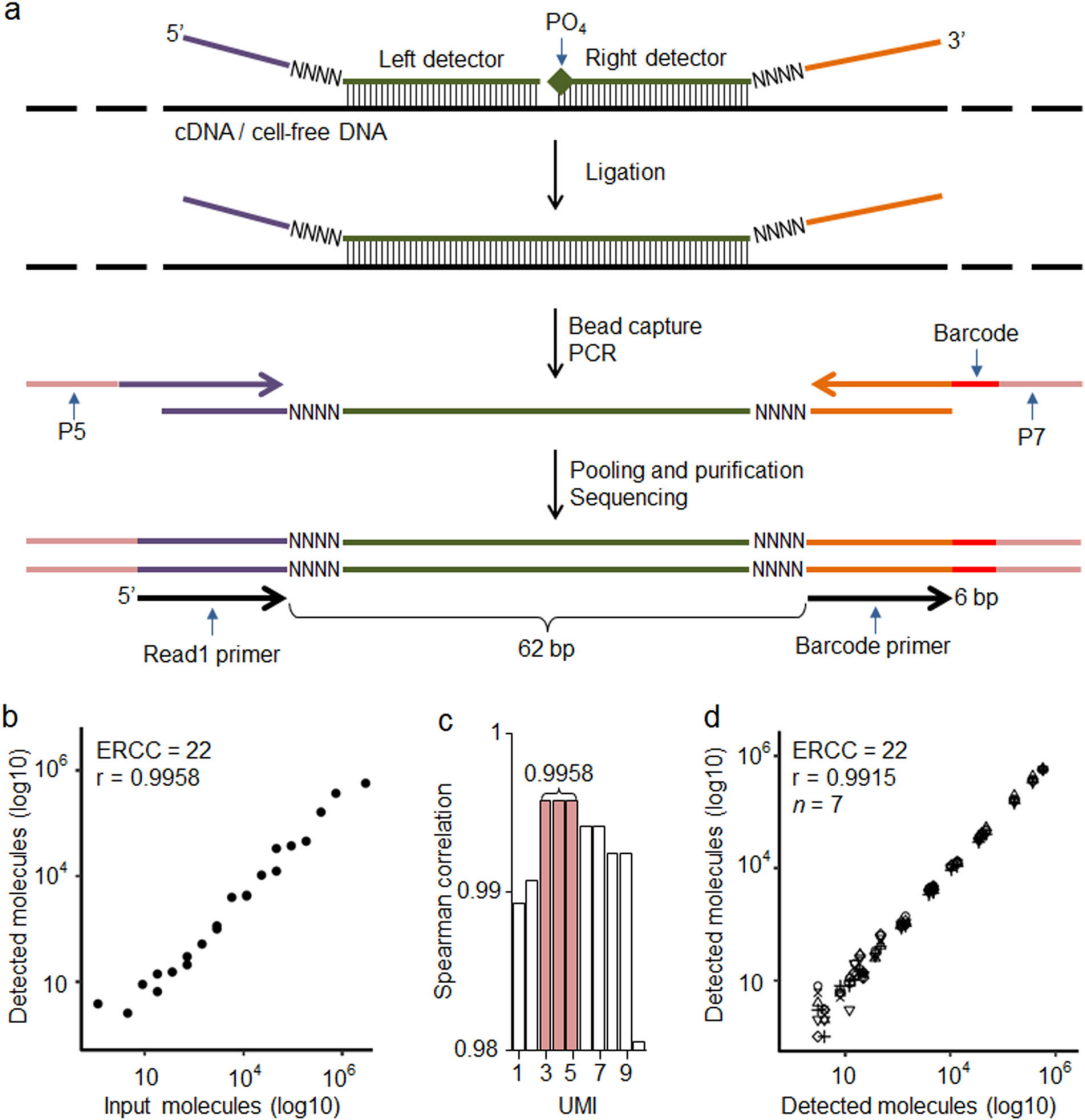
Principle and technical parameters of TAC-seq. **a** Schematic diagram of the assay to detect specific mRNA or cell-free DNA. Target-specific DNA oligonucleotide detector probes hybridize under stringent conditions to the studied cDNA or cfDNA. Both detector oligonucleotides consist of a specific 27-bp region (green), 4-bp unique molecular identifier (UMI) motif (NNNN), and universal sequences (purple and orange). The right detector oligonucleotide is 5′ phosphorylated. After rigorous hybridization, the pair of detector probes is ligated using a thermostable ligase under stringent conditions. Next, the ligated detectors complexed with the target region are captured with magnetic beads and PCR amplified to introduce sample-specific barcodes and other common motifs that are required for single-read NGS. **b** Spearman correlation analysis of the input and detected ERCC synthetic spike-in mRNA molecules at UMI threshold 4 (UMI = 4). UMI threshold is defined as the number of detected unique UMI sequences. For example, UMI = 4 indicates that a certain UMI motif is detected at least four times. UMIs are valuable only if the number of UMI combinations (8-bp UMI provides 65,536 variants, for example) is substantially larger than the sum of the target molecules in the studied sample. **c** Bar plot of Spearman’s correlation analysis of the ERCC input and detected molecules at different UMI thresholds. **d** Reproducibility of seven technical ERCC replicates (seven different icons on plot) of 22 spike-in molecules at UMI = 4

TAC-seq is a single-tube and ligation-based assay that allows precise biomarker quantification by the use of two UMI sequences in detector oligonucleotide probes. The studied mRNA and cfDNA molecules are uniquely identified using 54-bp-long target complementary sequence that is detected by two side-by-side located TAC-seq probes (Fig. 1a). Once stringent hybridization of the detector probes to the target occurs, a thermostable ligase is introduced, catalyzing the formation of a phosphodiester bond between the 5′-phosphate and the 3′-hydroxyl of two side-by-side detector probes. Next, ligated detector-target complexes are captured using magnetic beads and amplified by PCR (Supplementary Fig. 1), resulting in a ready-to-sequence library within a 3 h turnaround time. The risk of losing studied biomolecule is minimized by the dilution-free protocol in which ligated detector probes are captured, amplified, and identified by sequencing. To simplify the in silico design of specific TAC-seq probes and data analysis, we developed an online tool for designing the TAC-seq probes (http://nipt.ut.ee/design/) and a computational workflow software to enable data processing (open-source software link is in Methods and principle shown in Supplementary Fig. 2).

### Experimental evaluation of TAC-seq

First, we used External RNA Controls Consortium (ERCC) RNA spike-in controls to validate the technical sensitivity and accuracy of the TAC-seq method. Altogether, 22 spike-in sequences were assayed at various concentrations, ranging from 1 to 3 × 10^5^ molecules per reaction (Supplementary Table 1). The spike-in sequences were then detected with TAC-seq probes, sequenced and counted at different UMI thresholds. The analysis consistently demonstrated a high correlation (Spearman *r* > 0.99, Fig. 1b–c) between input and detected molecules for both relaxed and conservative (*n* > 1 molecules required) UMI thresholds.^18^ These results suggested that conservative UMI thresholds (*n* ≥ 3 molecules required in this case, Fig. 1c) are justified and applicable for high-coverage sequencing, in which the unfiltered read numbers are significantly higher than the UMI corrected outcome.^18^ With seven technical ERCC replicates, the average 1.5 × 10^6^ raw read-count per replicate dropped to 5.7 × 10^3^ after UMI correction, demonstrating a 102-fold average PCR redundancy at UMI threshold of 4 (*n* = 4) (Supplementary Table 1). Additionally, excellent reproducibility (Spearman *r* = 0.9915, Fig. 1d) among seven ERCC replicates was demonstrated.

### mRNA detection—precise molecule counting of endometrial receptivity biomarkers

**Fig. 2:**
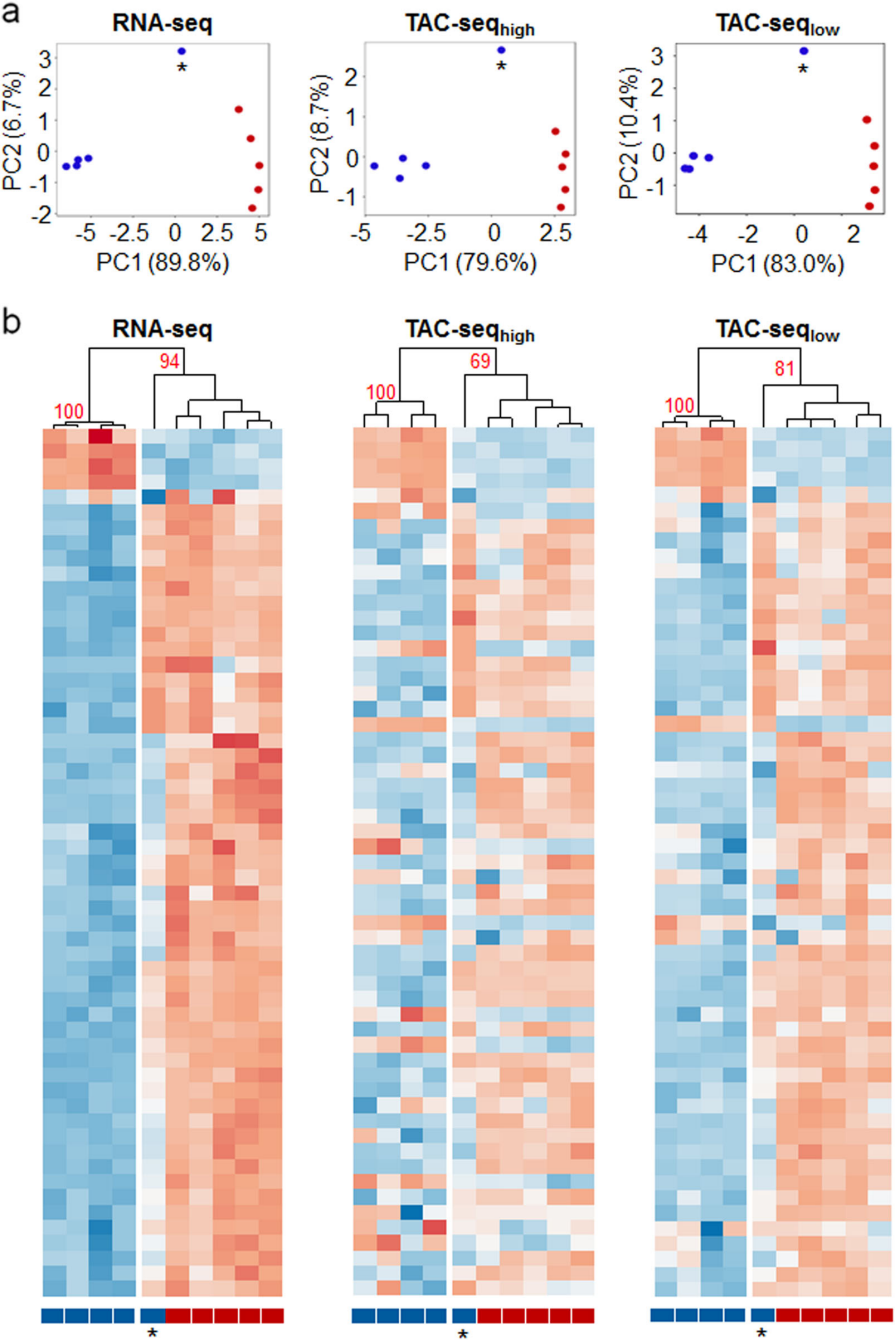
Comparison of the overall predictions for mRNA TAC-seq assay. **a** Principal component analysis of the full transcriptome RNA-seq, high-coverage TAC-seq and low-coverage TAC-seq of ten endometrial samples. The first principal component (PC1) describes most of the sample variability and correlates most with the receptivity status. Blue dots represent pre-receptive and red dots receptive human endometrial samples. One separate pre-receptive sample (indicated with an asterisk) represents the same sample that clusters differently in the heatmap analysis (below) and is, therefore, a potential biological outlier. **b** Heatmaps of the full transcriptome RNA-seq, high-coverage-, and low-coverage TAC-seq show the sensitivity to distinguish different endometrial samples according to their receptivity. One pre-receptive sample (indicated with an asterisk) shares the expression profile and clusters together with receptive samples in all three comparisons. Pre-receptive samples are labeled blue and receptive red. Detailed heatmaps are presented in Supplementary Fig. 3 together with housekeeping genes that demonstrate a lack of fluctuation of the pre-receptive and receptive biopsies. High-coverage TAC-seq data are presented at UMI = 2 and low-coverage data at UMI = 1 on PCA and heatmaps. Higher UMI thresholds in both high- and low-coverage approaches left low-expressed biomarker genes, like *APOD, EDN3* etc without reads, according to Supplementary Fig. 4. The data are plotted as row-wise scaled log-transformed counts per million (CPM) values. The samples are hierarchically clustered column-wise using Pearson correlation. The genes are ordered row-wise according to the RNA-seq clustering results using Euclidean distance. Fewer genes are found expressed with a low-coverage compared to RNA-seq and high-coverage TAC-seq

Next, we designed a transcriptome assay to analyze human endometrial linings. We targeted 57 endometrial receptivity mRNA transcripts that are potential biomarkers in reproductive medicine for testing embryo implantation compatibility.^19^ Ten endometrial biopsy samples were analyzed by TAC-seq and compared with the levels of selected 57 transcripts from full transcriptome RNA sequencing (RNA-seq) data. Principal component analysis of these data showed identical clustering of samples when applied to RNA-seq results and two different TAC-seq assays, carried out with high- (denoted as TAC-seq_high_, average 25.7 × 10^6^ reads per sample) and low-coverage (TAC-seq_low_, average 1.23 × 10^6^ reads per sample) sequencing (Fig. 2a). In these analyses, the first component described most of the sample variability (RNA-seq 89.8%, TAC-seq_high_ 79.6%, TAC-seq_low_ 83.0%) and distinguished the pre-receptive and receptive samples (*n* = 5 in both the groups, Fig. 2a), except for one outlier sample both in RNA-seq and TAC-seq analyses. The same sample grouping was confirmed by hierarchical clustering: four pre-receptive samples clustered together with high confidence (approximately unbiased (AU) probability of 100%), and receptive samples clustered together with one outlier sample in all datasets (AU_RNA_ = 94%; AU_TAC-high_ = 69%; AU_TAC-low_ = 81%) (Fig. 2b and Supplementary Fig. 3).

In both high- and low-coverage TAC-seq approaches all 57 differentially expressed transcripts were detected. However, high-coverage sequencing analysis revealed that 8-bp UMI causes technical limitation in molecule detection in the upper detection range. Six highly expressed genes met UMI-related saturation and were not precisely quantified (Supplementary Fig. 4). This technical limitation can be easily overcome by adding longer UMI nucleotides to detector oligonucleotides or by sequencing with lower coverage.

In low-coverage assay we expanded the assay to a 70-plex by adding housekeeping genes (*n* = 8) and selected ERCC spike-ins (*n* = 5). We observed that although the assayed housekeeping genes represented 47.4% of all unique reads in this assay, the biomarker-based clustering probability was still high (AU_TAC-low_ = 81%) and all of the targeted 57 biomarkers were detected (Fig. 2b). Based on these results, we suggest to include housekeeper genes (e.g., *CYC1, HMBS, SDHA*, and *TBP*) with low or moderate expression to low-coverage TAC-seq endometrium assay to ensure enough sequencing reads for all studied receptivity biomarkers.

### microRNA detection—differentially expressed molecules in endometrium

**Fig. 3:**
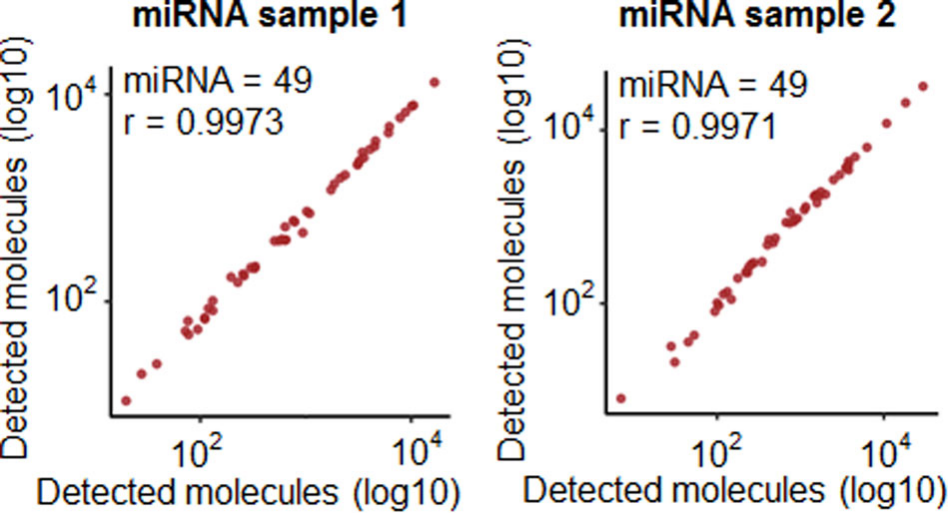
TAC-seq miRNA assay performance. Correlation plots of four miRNA sample technical replicates using TAC-seq assay at UMI = 4. miRNA sample 1 is on the left hand and has two replicates, one plotted on the *x*-axis and the other on the *y*-axis. The same with miRNA sample 2 on the right hand

To test the feasibility of TAC-seq with small non-coding microRNAs (miRNA), we selected 49 miRNAs from endometrial tissue, which were previously analyzed by small RNA-seq and covered highly variable expression levels from 10 to 4,712 counts per million. TAC-seq detected all 49 assayed miRNAs over 16 analyzed endometrial samples with high reproducibility (Spearman *r* > 0.997, Fig. 3) and showed high sensitivity in order to distinguish biologically different pre-receptive and receptive clinical biopsies in unsupervised clustering that were not detected by previous RNA-seq assay (Supplementary Fig. 5).

Due to the nature of miRNAs, short 20–24-bp target regions were detected using specific probes, and the TAC-seq protocol was modified accordingly (Supplementary Fig. 6). Additionally, an in silico prediction based on RNA-seq suggested that the selected set of miRNAs had nucleotide imbalance^20^ at positions one (*G* < 2%) and five (*C* < 3%), causing potential sequencing failure (Supplementary Fig. 7). The limitation was overcome using an adjusted spike-in probe that was designed to compensate for the two less-represented nucleotides at certain positions and therefore balance the whole sequencing run. The custom spike-in was later added to the sequencing reaction, resulting in a balanced nucleotide distribution and a high, 96% pass-filtering read rate (Supplementary Fig. 8). Although RNA-seq is convenient for miRNA profiling, our results confirm the previous findings^21^ that amplification bias reduction using UMI can enhance sensitivity. As a result, TAC-seq separates biologically different samples even in case of minor expression differences (Supplementary Fig. 5).

### Cell-free DNA detection—trisomy detection using controlled conditions

As the last test, we evaluated the potential of the TAC-seq method for NIPT, a widely used NGS-based clinical application. NIPT detects fetal trisomy based on the molecular counting of fetal cfDNA in maternal blood samples. We designed a proof-of-principle trisomy 21 detection assay to demonstrate the applicability of the TAC-seq method for absolute cfDNA molecule counting to determine fetal trisomy at different experimentally controlled ‘‘fetal fraction’’ levels. In the following in vitro aneuploidy detection experiment, we mixed different proportions of gDNA from a chr21 trisomy cell line with a known normal control gDNA. Different proportions of fetal trisomic cfDNA were created to imitate the range of fetal fraction in mothers’ circulating cfDNA. gDNAs were sheared by sonication to mimic 160–180 bp cfDNA, mixed to yield 5–30% trisomy proportions and hybridized with TAC-seq detector probes that were designed to target reference chromosomes (chr2 and chr3) and studied chr21.

**Fig. 4:**
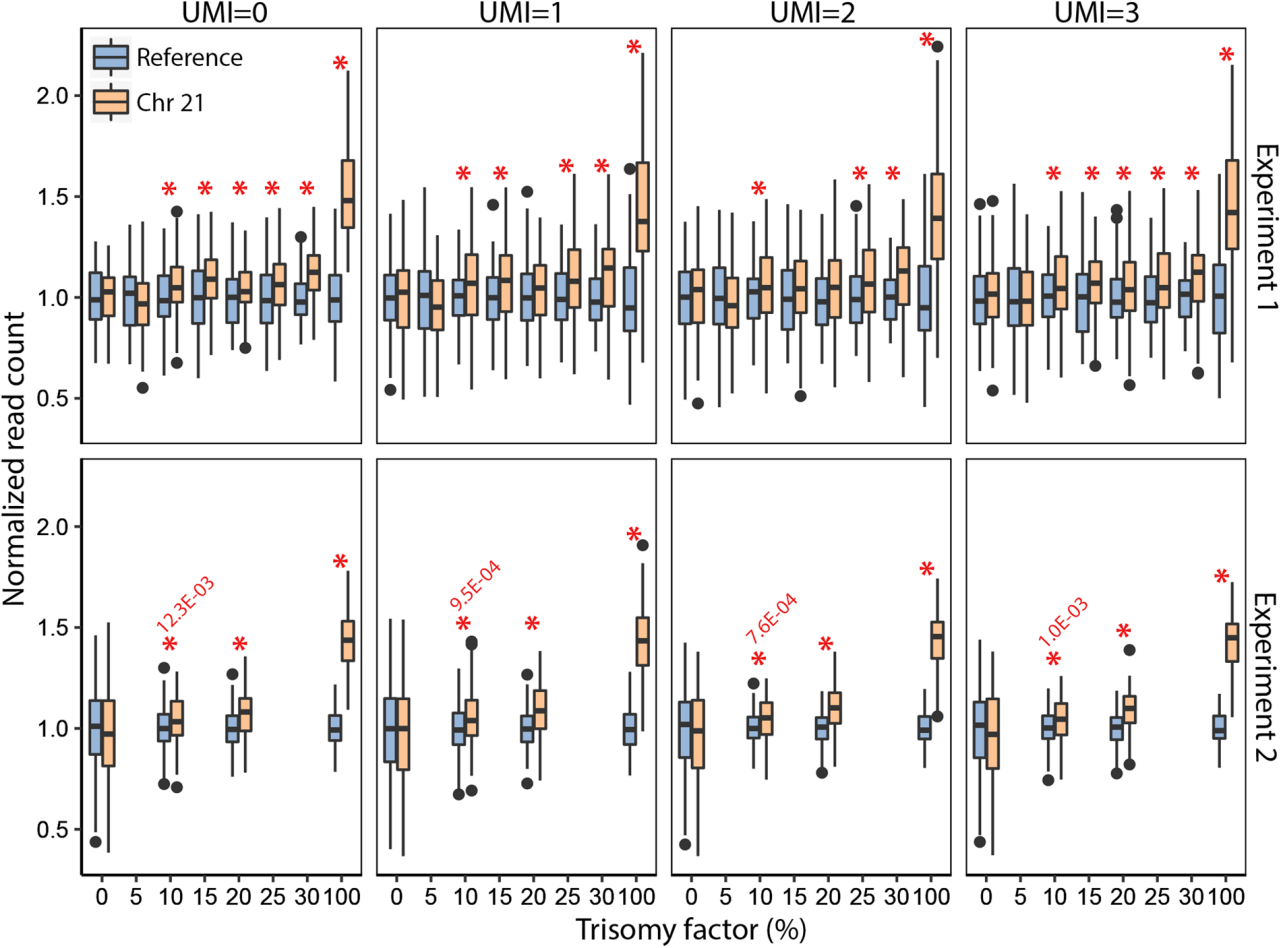
Trisomy detection under in vitro conditions. Boxplots over applied UMI thresholds of normalized molecule counts (*y*-axis) of trisomy TAC-seq experiments indicates a positive correlation between the trisomy factor (*x*-axis, trisomic cell proportion) and chr21 counts. Experiment 1, upper four plots, involved 114 loci along chr2 and chr21. One biological replica is depicted. Experiment 2, lower four plots at various UMI thresholds, involved extended TAC-seq probe set (in total 224 probes) along chr2, chr3, and chr21. The red asterisks indicate significant reference chromosome(s) and chr21 read-count-based differences between studied samples (*p* < 0.05, one-tailed Welch’s *t*-test)

The detection of trisomy 21 demonstrated a significant difference at the lowest 10% ‘‘fetal cfDNA fraction’’ (Fig. 4), which improved even further in case of every additional 5% increase in the ‘‘fetal fraction.’’ The same result was confirmed by whole-genome re-sequencing NIPT assay for the same samples^22^ (Supplementary Fig. 9). Replication TAC-seq experiment with extended probe set demonstrated significant (*p* = 7.6 × 10^−4^) excess of chromosome 21 molecules at 10% fetal fraction level, applying UMI = 2 threshold (Fig. 4).

## Discussion

Combining high sensitivity and flexibility of NGS with cost-efficient and precise quantification of targeted methods can enable robust detection of specific nucleic acid biomarkers indicative of (patho)physiological conditions. TAC-seq is an advanced ligation-based NGS method that differs from existing ligation-PCR assays such as MLPA^6^, MLPA-seq^5^, TempO-Seq^4^, RASL-seq^7^ and DANSR.^8^ The major advantage of TAC-seq is ability to detect the number of original molecules of transcriptomic biomarkers such as mRNA and miRNA, and genomic loci from cfDNA. Precise molecule counting is achieved by integrated UMI or ‘‘molecular barcode’’ motif^12^, which decreases the quantitative and random bias introduced by in vitro replication steps. Using UMIs removes PCR duplicates, reducing one of the major NGS-specific technical biases and improves the accuracy of NGS.

We detected very high sensitivity correlation over 22 analyzed ERCC spike-in input and detected molecules (Spearman *r* = 0.9958 on Fig. 1d) with high-coverage, ensuring that each UMI coverage was 102×. Based on the coverage, we are confident that very few UMIs have been missed and, therefore, this outcome is reliable. However, systematic difference appears between lowly- and highly expressed targets with the number of high copy-number molecules is underestimated (see top-four ERCC spike-ins in Supplementary Table 1). This is explained by the length of UMI sequences, causing ‘‘technical saturation.’’ Eight nucleotide UMIs used in our study have 65 thousand possible sequences, which is well suitable for cfDNA-based trisomy detection as copy numbers of cfDNA in 10 ml of blood remains <5000.^23,24^ The same applies to TAC-seq expression applications if lower RNA concentrations are used. Alternatively, it is possible to extend UMI sequences in both detector probes from current 8 to 12 nucleotides, ensuring 16.7 million possible combinations. At the same time we are aware that introducing significantly longer and random UMI strings into detector probes may increase the probability of probes self-pairing and unspecific ligation. However, UMI-related issues such as ‘‘saturation’’ and replication-caused novel, ‘‘phantom’’ UMIs^25^ that should be taken into account in assay design and data analysis.

TAC-seq was designed while keeping in mind the main prerequisites of genetic testing laboratories—sensitivity, robustness, and cost-efficiency. Sensitivity and molecule counting by UMI was discussed above. Robustness is ensured by single-tube protocol to minimize the risk of allelic drop-out. Furthermore, the approach is dilution-free, meaning that analyzed biomarker molecules together with ligated detector probes are captured and identified by sequencing. The latter is crucial in liquid biopsy samples where each locus is represented only by some thousands of copies. If ligation-based assay with specific probes is used then probe hybridization-compatible target cfDNA copy-number reduces 25% due to the short length of cfDNA (180 bp) as the locus is not detected if it locates closer than 25 bp to cfDNA fragmentation site.

TAC-seq detects mRNA biomarkers through oligo-T primed cDNA synthesis (poly-A selection) that reflects the analysis of active transcriptome. It differs from TempO-Seq^4^ where recently described SplintR ligase^26^ for RNA/DNA hybrid is used to detect any, even fragmented RNA targets by specific detector oligonucleotides. In addition, SplintR ligase optimum working temperature is up to 37 °C that may limit the specificity of formed RNA/DNA probe complexes prior ligation. In contrast, TAC-seq uses thermostable *Thermus aquaticus* (*Taq*) DNA ligase^27^ that enables specific hybridization and ligation at temperature above 45 °C. Based on this property of *Taq* DNA ligase, we have carried out first specific probe-target hybridization at 60 °C and thereafter introduced ligase to join the proximity annealed strands at the same temperature.

As sequencing contributes to the majority of the NGS cost, it is critical to apply library preparation that supports low-coverage sequencing in routine NGS clinical applications. Cost-effectiveness of TAC-seq is ensured by off-the-shelf reagents and the usage of common instruments in genomic laboratory, such as standard thermocycler and benchtop NGS sequencer. The running cost of TAC-seq is only a fraction of the cost for commonly used NGS applications like whole-genome sequencing for NIPT or RNA-seq for mRNA and miRNA analysis. Set-up cost of TAC-seq depends on number of studied loci due to the need of specific detector oligonucleotides (Supplementary Fig. 10). Consumables and their approximate prices are listed in Supplementary Table 2 and explained in Methods. Based on our in-house library preparation and sequencing, the total reagent cost for miRNA profiling and cfDNA analysis is less than 30 EUR per sample and 26–40 EUR per sample for mRNA biomarker analysis, depending on the sequencing depth. Therefore, TAC-seq has the potential to become a cost-effective alternative for routine NIPT after clinical studies or for detecting the levels of transcriptome biomarkers.

TAC-seq probe specificity is ensured by 54-bp-long region on mRNA and gDNA. We developed automated mRNA probe design software (http://nipt.ut.ee/design/) without restrictions in usage and described in Supplementary Methods) that automates probe design procedure and provides highly specific oligonucleotide sequences with common motifs that are ready for synthesize. Probe design for miRNA molecules is even more straightforward and does not require special software (see Supplementary Fig. 6). Another simplification that makes NGS as a choice of detection method is user-friendly data analysis. Small-scale NGS data analysis does not demand powerful computing resources. For this, we provide user-friendly personal computer software for small-scale TAC-seq data analysis and open-source code for intense analysis (link in Methods). The NGS ‘‘big data’’ limitation has been overcome by simple analysis pipeline. Most resource-demanding raw data processing after sequencing is done by Illumina cloud-computing environment. Following TAC-seq analysis is based on text-file manipulations eliminating the need of sequencing read mapping, making it possible to perform NGS analysis in personal computer (see details on Methods).

Applied UMI threshold depends on the type of application and sequencing depth. cfDNA analysis bases on the expectation that all studied loci are represented in relatively similar copy-number. If number of PCR cycles is optimized to avoid excess amplification, we recommend to use UMI = 1 or UMI = 2 threshold (Fig. 4). Transcriptome biomarker analysis is faced to diversity in original molecule counts (Supplementary Fig. 4). The differences in transcriptome determine optimal UMI threshold according to lowly expressed molecules. Too stringent threshold filters out lowly expressed biomarkers.

In conclusion, we have developed a highly sensitive and parallel method to count accurately the number of nucleic acid biomarker molecules in studied samples. Our proof-of-principle study demonstrates that TAC-seq has similar sensitivity to golden standard RNA-seq method in case of mRNA and miRNA application, and can successfully detect the excess of cfDNA molecules (indicative of chromosomal trisomy) in cfDNA-like material. TAC-seq is automation-compatible method that is designed to overcome ligation- and NGS-based limitations in genetic testing laboratory. Although all presented applications need careful clinical validations before they can be utilized, the described method is the base for further specialization and optimization to provide advanced DNA and RNA biomarker analysis tools and thereby improve reach and quality of corresponding research and healthcare applications.

## Methods

### Studied samples

This study was approved by the Research Ethics Committee of the University of Tartu (246/T-21 and 221/M-31). Endometrial biopsies for RNA assays were collected from healthy volunteers and written informed consent was obtained from all participants. Genomic DNAs from GM01359 (47,XY, +18) and GM04616 (47,XX, +21) cell lines (NIGMS Human Genetic Cell Repository, Coriell Institute of Medical Research) were extracted using the DNeasy Blood and Tissue kit (Qiagen). To mimic cfDNA, genomic DNA was fragmented to 150–200 bp using Covaris M220 Focused-ultrasonicator (Thermo Fisher).

Detail description of biomarker selection, TAC-seq probe design, library preparation protocols, and TAC-seq sequencing are described in Supplementary Methods.

### TAC-seq data analysis

ERCC spike-in reads were trimmed to construct length of 88-bp and demultiplexed by barcodes (6-bp) allowing 1 mismatch. Demultiplexed reads were further trimmed to length of 62-bp and 4-bp of UMI at the end of the read was inserted after 4-bp of the UMI at the start of the read. Reads with UMI that contained unallocated nucleotides, were discarded. Resulting reads per sample were demultiplexed again using target regions of the genes (54-bp) allowing up to five mismatches. Total read counts, unique molecule counts and Pearson and Spearman correlations were calculated at different UMI thresholds. Due to the fact that Pearson method assumes linear correlation and, therefore, resulted in insensitive correlations at high UMI thresholds, we chose Spearman correlation as it proved to work correctly in case of very high and very low molecule concentrations.

Gene expression reads were processed as described above. To reduce potential sequencing error accumulating at UMI motif, only reads appearing at least twice were counted as unique molecules (UMI = 2). Each sample was normalized to CPM using edgeR (version 3.18.1) package in R (version 3.4.1) and log-transformed using the log10 (CPM + 1) transformation to reduce skewness. In addition to biomarker genes, both high- and low-coverage TAC-seq libraries included spike-in molecules. Furthermore, the low-coverage library included eight housekeeping genes, which were taken into account in CPM normalization. The normalization procedure was based on the published formula that was further adjusted for read-count data.

Genomic DNA sequencing data quality control and pre-processing were performed as described above. Loci that were 1.5 interquartile ranges (IQRs) below the first quartile or above the third quartile were called as outliers and removed. As we constantly detected slightly higher molecule counts in chr2 compared to chr21 in euploid samples, chromosome-specific molecule counts were applied. For that, mean molecule counts of chr2 and chr21 (~1.081 at UMI threshold 2) using the euploid samples were calculated and used for normalization. User-friendly software was developed to enable TAC-seq data processing in end-user’s personal computer or in Linux environment.

### Code availability

Open-source software with installation instructions are available at https://github.com/cchtEE/TAC-seq-data analysis and shown schematically in Supplementary Fig. 2.

### Set-up and running cost of TAC-seq

Targeted assay set-up needs two specific probe oligonucleotides for mRNA and cfDNA analysis. The price we got was 27 EUR per detector pair, resulting in 800 EUR in total for 30 loci analysis, for example (Supplementary Fig. 10a). miRNA detection needs only one non-phosphorylated specific detector per molecule of interest (Supplementary Fig. 6), lowering the set-up cost to 200 EUR in case of 30 loci. Reagent costs, using off-the-shelf consumables, show that cDNA synthesis for mRNA assay is 0.6 EUR per entire sample that is more cost-effective than miRNA cDNA synthesis (3.2 EUR/sample) that needs more manipulations. Following ligation, purification and quality control cost 5.4 EUR for mRNA, miRNA, and cfDNA applications (Supplementary Table 2). Sequencing contributes majority of the analysis reagent price, being in the range of 20–34 EUR per sample, depending on the application and sequencing depth (Supplementary Fig. 10b). Based on our in-house library preparation and sequencing protocol, the total reagent cost for miRNA profiling and cell-free DNA analysis is <30 EUR per sample and 26–40 EUR per sample for mRNA biomarker analysis.

## Supplementary information

Supplementary information

## Data Availability

Supplementary sequencing data are available in Gene Expression Omnibus database under accession codes GSE98386 and GSE110110, and in Sequence Read Achieve under accession code SRP132266.

## Supplementary information

**Supplementary information** accompanies the paper on the *npj Genomic Medicine* website (10.1038/s41525-018-0072-5).
