# Supplementary material for: TAC-seq: targeted DNA and RNA sequencing for precise biomarker molecule counting
Source: NPJ Genom Med. 2018 Dec 18;3:34. doi: 10.1038/s41525-018-0072-5 (PMC6299075; doi:10.1038/s41525-018-0072-5)
Supplement: Supplementary file 1 — Supplementary information [file 41525_2018_72_MOESM1_ESM.pdf]

Supplementary materials

Supplementary methods

Biomarker selection and TAC-seq probe design

The biomarkers of endometrial receptivity were selected based on our previous publication.[19] Briefly, nine studies including a total of 164 endometrial samples from fertile women were included in a meta-analysis using the Robust Rank Aggregation method. In the current study, we used the 57 mRNAs identified as potential endometrial receptivity biomarkers for distinguishing the pre-receptive and receptive endometrial samples. A pair of TAC-seq detector DNA oligonucleotide probes (left detector and right detector) was designed for every targeted gene using the special TAC-seq probe design software (http://nipt.ut.ee/design/). All of the oligonucleotides used in this study are listed in Supplemental Table 3. Both the left and right detectors consisted of a specific sequence (27-bp), an UMI (4-bp) and a left universal sequence or right universal sequence. Each detector pair targeted the coding sequence in the Consensus Coding Sequence Set (CCDS). For genes without CCDS, the most likely transcript was chosen manually from the Ensembl 87 database. Selection of the target sequence was based on two criteria. First, the adjacent 14-bp, 7-bp from both detector probes around the ligation site, had to be unique against human cDNA (GRCh38) to minimize the likelihood of non-specific hybridization. Next, the unique sequences were ranked according to the distance from the 3'-end of the transcript. Routine genetic testing detectors were preferentially designed close to transcript's 3'-end to minimize the effect of possible RNA degradation caused by sampling and handling if poly-A at the mRNA 3'-end is used for cDNA priming. Additionally, detector-specific regions were filtered by GC-content to determine the optimal melting temperature. The overall GC-content of a probe had to be between 40–60%, and the GC-content of the adjacent ends (4-bp) was up to 50%. Additionally, detector oligonucleotides with inter- or intra-complementarity issues were excluded from the selection. Although mRNA's 3'-ends were targeted in this study, the software has an option to design TAC-seq detectors close to the transcript's 5'-end, if required. The ERCC spike-in 22 detectors were designed based on the above description close to their poly-A tails.

For the TAC-seq miRNA assay, 49 miRNAs showing stable expression values (standard deviation/mean count per million (CPM) <0.5) within a study group were chosen according to previously published small RNA sequencing data.[19] One specific 20–24-bp detector oligonucleotide was designed per each selected miRNA ('Specific detector' in Supplementary Fig. 6). Eight UMI nucleotides and a common sequence were added to each specific detector probe. The right detector oligonucleotide is universal for all miRNAs, consisting of two common sequences and a 5' phosphate to enable ligation.

Chromosome 2 and 21 loci were selected from the k-mer http://bioinfo.ut.ee/NIPTMer/programs/lists/ database (converted to text files with glistquery http://bioinfo.ut.ee/NIPTMer/programs/glistquery where k-mers overlapping known polymorphisms (dbSNP build ID 149) were first removed and the remaining candidates were used as an input for BLAST 2.4.0+ (task blastn) with database version GRCh38 (GCA_000001405.15). All reads with more than one exact match were removed, following the concatenation of overlapping regions. The regions were converted to sequences with UCSC Genome Browser Gateway. Altogether, 114 specific detector pairs over the studied chromosomes 2 and 21 were selected according to the above-described design, ensuring equal coverage over the entire chromosome.

ERCC mRNA library preparation

Non-skirted low profile PCR Strip Tube Plates (Thermo Fisher) were used with domed cap strips (Thermo Fisher). ERCC Spike-In Mix 1 (Life Technologies) was first diluted 10× and then additionally 100× with water. Aliquots, each containing 1.3 µl of 1,000× dilution, were stored at -70°C until use. Next, 199 µl of water was added to 1.3 µl aliquot and mixed. 1 µl of diluted ERCC spike-in content (Supplementary Table 1), serving as a template for each individual library was added to 2 µl of denaturation buffer, containing 5 mM Tris-HCl (pH 7.0) (Sigma-Aldrich), 1 mM dNTP mixture (Thermo Fisher), 400 nM Oligo-T30 primer and 0.05% Triton X-100 (Sigma-Aldrich). Reaction was mixed by pipetting and centrifuged briefly. RNA was denatured by 1 min at 80°C and immediately placed on ice. After that, reverse transcriptase (RT) master mix containing 100 mM Tris-HCl (pH 8.5) (Sigma-Aldrich), 2.5 M betaine (Sigma-Aldrich), 150 mM KCl (Sigma-Aldrich), 10 mM DTT

(Sigma-Aldrich), 15 mM $MgCl_2$ (Sigma-Aldrich), 4 U RiboLock RNase inhibitor (Thermo Fisher) and 20 U Maxima H Minus Reverse Transcriptase (Thermo Fisher) was prepared. The master mix was vortexed and briefly centrifuged. 2 µl of RT master mix was added to previously denatured RNA (3 µl). All RT pipetting steps were performed on ice. cDNA synthesis was performed by 30 min at 42°C, following 5 min at 85°C for RT inactivation.

Twenty two TAC-seq detector pairs targeting ERCC spike-in molecules were previously mixed together from 100 µM stock solutions, creating a '100 µM' oligo pool. The oligo mixture was diluted to 5 µM by water and stored at -20°C. Once cDNA synthesis was completed, 1 µl of 5 µM TAC-seq detector mixture was added to RT mixture. The content was mixed on vortex and centrifuged briefly. Strip tubes were placed on thermocycler, cDNA denatured for 1 min at 98°C, followed by 1 h at 60°C to enable specific cDNA and TAC-seq probe hybridization. After hybridization, thermostable ligase reaction mixture was added. To keep a constant hybridization temperature (60°C), the cycler lid was opened and strip caps were removed. 5 µl of Taq DNA ligase master mix, containing 2× Taq DNA ligation buffer (New England Biolabs, NEB) and 1 U Taq DNA ligase (NEB) were added to each individual reaction tube and mixed by pipetting. The strip tubes were not removed from 60°C thermocycler to avoid self- and mispairing of TAC-seq probes. Ligation reaction was stopped after 20 min incubation by placing the reaction tubes on ice.

15 µl of mixture consisting of Dynabeads MyOne Carboxylic Acid beads (2 µl) (Thermo Fisher) and 13 µl of capture buffer (30% PEG-6000, 2 M NaCl, 5 mM Tris-HCl (pH 7.5), 10 mM EDTA and 0.02% Tween-20 (all chemicals from Sigma-Aldrich)) was added to ice-cooled ligated sample. The content was mixed by vortex. Capture was carried out for 10 min at room temperature. After that the tubes were placed on DynaMag-96 Side Magnet (Thermo Fisher) holding 8-well strip tubes on VersiPlate Frame (Thermo Fisher). Supernatant was removed after 3 min incubation on the magnet. The beads on magnet were washed once with 50 µl of fresh 80% ethanol. Ethanol was removed by pipetting, and the clean pellet, without ethanol drops, was dried for 2 min. Once beads were dry, strip tubes were removed from the magnet and 18 µl of PCR master mix was added directly to the beads. We have also successfully performed magnetic bead capture prior PCR without ethanol washing to avoid the risk of over-drying the bead. In the latter case, the supernatant should be removed

completely. PCR master mix contained 1× proofreading HOT FIREPol Blend Master Mix (Solis BioDyne, Tartu, Estonia) and 250 nM TAC-seq left primer. In addition to universal TAC-seq left, 16 different TAC-seq barcoded oligonucleotides were used to introduce a 6-bp barcode to each studied sample (Supplementary Table 3). For that, 2 µl of 5 µM TAC-seq barcoded 1–16 primers were added individually to each PCR reaction. Strip tubes were closed with clean domed caps, mixed on vortex until beads were completely re-suspended. The ERCC spike-in reaction was incubated at 95°C for 12 min, followed by two cycles of 95°C for 20 s, 57°C for 60 s and 72°C for 20 s. In addition, 16 cycles of 95°C for 20 s, 65°C for 20 s and 72°C for 20 s with a final extension at 72°C for 1 min using the default ramp speed of the T100 cycler (Bio-Rad) were performed. PCR products were pooled together into 1.5 ml tube. The tube with pooled sample was placed on magnet to remove carboxylated beads before the following column purification. Clear supernatant was purified with DNA Clean & Concentrator-5 column (Zymo Research) and eluted with 50 µl of elution buffer (EB). The library was size-selected using AMPure XP beads (Beckman Coulter) in a single-step selection to reduce 81 bp linear PCR double-stranded by-product (Supplementary Fig. 1). 50 µl beads were added to 50 µl of the purified PCR product, incubated for 5 min at room temperature and captured by a magnet for 3 min. After incubation on magnet, the supernatant was discarded and the remaining beads were centrifuged at 500×*g* for 10 s. After centrifugation, the beads were placed again on the magnet and all remaining supernatant was removed. The beads were eluted directly without ethanol washing in 25 µl of EB and incubated for 1 min at room temperature. AMPure XP bead elution has almost 100% efficiency even without previous ethanol wash. Finally, the eluted library was transferred to a clean tube after 1 min incubation on the magnet. The 180 bp library (Supplementary Fig. 1a-d) was visualized on a TapeStation High Sensitivity D1000 ScreenTape (Agilent Technologies) and quantified using KAPA Library Quantification Kit (Roche).

Clinical sample mRNA library preparation

mRNA biomarker libraries for endometrial receptivity testing were prepared as described above with the following modifications. Total-RNA samples with RIN values 7.7–9.6 (quantified by Qubit (Invitrogen)) were diluted to concentration of 90 ng/µl and 1 µl of this was used for library

preparation. RT master mix contained 1 µl of 1:50,000 of ERCC RNA Spike-In Mix 1 (Life Technologies) dilution for technical normalization. Altogether 64-plex TAC-seq probe set, containing 57 biomarker genes [19] and seven ERCC spike-ins (ERCC-00085; 00170; 00019; 00131; 00092; 00108 and 00004) were used to generate a library for high-coverage analysis. The low-coverage analysis was performed using 70-plex, containing 57 biomarker genes,[19] five ERCC spike-ins (00131; 00108; 00092; 00019 and 00004) and eight housekeeping genes (*ACTB*, *GAPDH*, *YWHAZ*, *PPIA*, *CYC1*, *HMBS*, *TBP* and *SDHA*). 5 µM detector oligonucleotide mixtures from 100 µM stocks were created as described above. PCR was performed using in total 12 cycles, following 2+10 principle (described in details above) for both high- and low-coverage libraries.

microRNA library preparation

miRNA profiles were analysed from endometrial total-RNA. 3' ligation was carried out overnight in 5 µl volume. The reaction contained 100 ng of total-RNA, 1× RNA T4 RNA Ligase Reaction Buffer (NEB), 20 U RNase inhibitor (Thermo Fisher), 10% PEG-8000 (NEB), 100 nM adenylated 3' linker and 40 U T4 RNA ligase 2 (truncated, NEB). After ligation, the free ligation adapter was removed by adding 0.5 µl 5'-Deadenylase (25 U/µl, NEB) and 0.5 µl Lambda exonuclease (5 U/µl, NEB) and incubated 10 min at 37°C, followed by 10 min at 75°C. cDNA was synthesized after adding 0.4 µl 100 mM DTT (Invitrogen), 0.4 µl 2 M KCl (Sigma-Aldrich), 0.4 µl 10 mM dNTPs (Thermo Fisher), 0.4 µl RNase inhibitor (Thermo Fisher), 0.2 µl 10 µM micro RT biotin primer and 0.2 µl Maxima H Minus Reverse Transcriptase (200 U/µl, Thermo Fisher) which were mixed into one 2 µl master mix. cDNA incubation was carried out for 15 min at 50°C, followed by 5 min at 80°C. Unbound primers were removed by adding 1 µl Exonuclease I (20 U/µl, Thermo Fisher) and incubating for 10 min at 37°C and 5 min at 95°C. 1 µl of 5 µM TAC-seq detector mixture, containing miRNA-specific left detectors and miRNA universal 5' phosphorylated detector oligonucleotide (Supplementary Fig. 6), was added to previous 9 µl product and incubated first for 2 min at 98°C to denature the template and probes and then for 1 h at 60°C. After the hybridization, thermostable ligase reaction mixture was added on thermocycler, keeping a constant (60°C) hybridization temperature. The cycler lid was opened and strip caps were removed. 5 µl of Taq DNA ligase mixture, containing 2× Taq DNA ligation buffer

(NEB) and 1 U Taq DNA ligase (NEB) was added to each individual reaction tube and mixed by pipetting. Ligation was stopped after 20 min incubation by placing reaction tubes on ice. 3 µl of Dynabeads MyOne Streptavidin C1 beads (Invitrogen) were washed according to protocol and suspended in 15 µl recommended B&W buffer. The beads were added to ligated product on ice, mixed well by pipetting and incubated for 10 min at room temperature. After capturing the beads on magnet for 1 min, the supernatant was removed and the beads were washed once with B&W buffer. TAC-seq ligated detectors were amplified as described above using 2+18 cycles of PCR. The designed miRNA library is 170 bp (Supplementary Fig. 1e).

Cell-free DNA library preparation

10 ng of acoustically sheared (Covaris) cell-free-like genomic DNAs were combined to create excess rates of chr21 above euploid level, mimicking the extra 5–30% of fetal cfDNA fractions. 100% fraction is the GM04616 cell line's DNA with trisomy 21. Each concentration was performed as duplicate. Samples were pipetted into strip tubes, adding 1 µl of 5 µM TAC-seq detector oligonucleotide mixture and 1 µl 10× hybridization buffer, containing 100 mM Tris-HCl (pH 7.5), 500 mM KCl, 0.2% Tween-20 and 0.1 mM EDTA. The final hybridization volume was 12 µl. The content was mixed by vortexing and centrifuged briefly. Strip tubes were placed on thermocycler, mixture denatured for 2 min at 98°C, followed by 2 h at 60°C for hybridization. After hybridization, thermostable ligase reaction master mix was added on thermocycler, keeping constant (60°C) hybridization temperature. Subsequently, 2.5 µl of Taq DNA ligase master mix, containing 1.5 µl 10× Taq DNA ligation buffer (NEB) and 1 U Taq DNA ligase (NEB) was added to each individual reaction tube and mixed by pipetting. Ligation reaction was stopped after 20 min incubation by placing reaction tubes on ice. 25 µl of previously combined Dynabeads MyOne Carboxylic Acid beads (3 µl) (Thermo Fisher) and 22 µl of capture buffer as described above was used for capture in this assay. Ligated TAC-seq detectors were amplified as above described using 2+19 PCR cycles.

MicroRNA spike-in preparation

Custom miRNA spike-in was prepared with PCR using 76 bp synthetic ‘miRNA spike-in’ oligonucleotide, ‘TAC-seq left’ and ‘miRNA spike-in right primer’ (Supplementary Table 3). PCR was carried out in 100 µl volume containing 20 µl HOT FIREPol Blend Master Mix (Solis BioDyne), 1 µl 100 nM miRNA spike-in DNA oligonucleotide as a template, 1 µl 100 µM TAC-seq left and miRNA spike-in right primers. The reaction tube was incubated at 95°C for 12 min, followed by two cycles of 95°C for 20 s, 57°C for 60 s and 72°C for 20 s. In addition, 8 cycles of 95°C for 20 s, 65°C for 20 s and 72°C for 20 s with a final extension at 72°C for 1 min were used. The product was purified by column and quantified by KAPA Library Quantification Kit (Roche).

Reference sequencing and data analysis

Total-RNA samples with concentration at least 200 ng/µl and RIN >8 were used for endometrial receptivity cDNA library construction. Libraries were generated from ~1 µg of total-RNA using TruSeq Stranded Total RNA (Illumina) protocol. Libraries were normalized, pooled and sequenced by Illumina HiSeq2500 instrument producing 100 cycles paired-end reads. The RNA-seq data was analyzed as previously described.[19] Heatmaps of the results were generated using the ‘pheatmap’ package implemented in R. For plotting, CPM values provided by edgeR were log-transformed, using the transformation log(CPM+1) to facilitate graphical presentation of the results.

Previously published small RNA sequencing data, containing the same RNA samples as in the miRNA TAC-seq experiments, was used. Briefly, libraries were constructed following a TruSeq Small RNA Library Preparation Guide (Illumina). 1 µg of small RNA fraction isolated from endometrial tissues was used as an input. Libraries were sequenced by Illumina HiSeq 2500 instrument producing 50 bp single-end reads. The RNA-seq data was analyzed as previously described.[19]

Sheared genomic DNA samples with concentration 5 ng/µl were used to generate cfDNA libraries as described elsewhere but using 12 cycles of PCR. Libraries were quantified by Qubit HS assay (Thermo Fisher), brought to the uniform concentration and pooled. The pooled library quality was estimated using a TapeStation High Sensitivity D1000 ScreenTape (Agilent Technologies) and sequenced by Illumina NextSeq 500 instrument producing 85 bp single-end reads. A previously

described method was used for the analysis including mapping of sequencing reads to the reference genome, calculating the coverage of each region in the genome, GC correction, calculating the mean and standard deviation of the reference population and the sample. Finally, risk for aneuploidy was estimated by calculating Z-score, as well as additional ZZ-score, BM (bin median) and OM (other median). Trisomy is called if Z-score is ≥ 3, ZZ-score is ≥ 3, BM is ≥ 1.5 and OM is < 1 (Supplementary Fig. 9).

TAC-seq sequencing

The ERCC spike-in library was sequenced by Illumina NextSeq 500 high output 75 cycles kit using 2 pM library concentration. The library was sequenced using 90-bp single-read protocol that was primed by Illumina Read1 (HP10) primer. The entire construct was 90-bp. The second, high-coverage mRNA biomarker set was sequenced with configuration identical to the one described above. In both libraries, particularly in receptivity biomarker assay, 2-channel Illumina SBS technology caused reduced level of cluster quality due to a common 20-bp motif (an extremely low-diversity region) at construct 62–82-bp site (Supplementary Fig. 8a). 4-channel SBS was used with the same library and 90-bp read using MiSeq (Illumina) instrument (data not shown) without any improvement. Following custom barcode sequencing primer was designed and used for low-coverage mRNA biomarker assay, analyzed by MiSeq Reagent Kit v3 in 14 pM library concentration. Custom barcode primer avoided the low-diversity common region and significantly improved the outcome, increasing the chastity filter (pass-filter) per cent from previous 67% to 93% (Supplementary Fig. 8b-d). In total 62-bp Read1 and 6-bp barcode (index) nucleotides were sequenced. miRNA library was sequenced by NextSeq 500 high output 75 cycles kit and 2 pM library concentration using LNA custom barcode primer. The Read1 length was 32-bp plus 6-bp barcode. Cell-free DNA library was analyzed by NextSeq500 instrument, using custom LNA barcode primer, 1.8 pM loading concentration, 62-bp for Read1 and 6-bp for barcode. The data have been deposited under GEO accession number GSE98386 and GSE110110 and SRP accession number SRP132266.

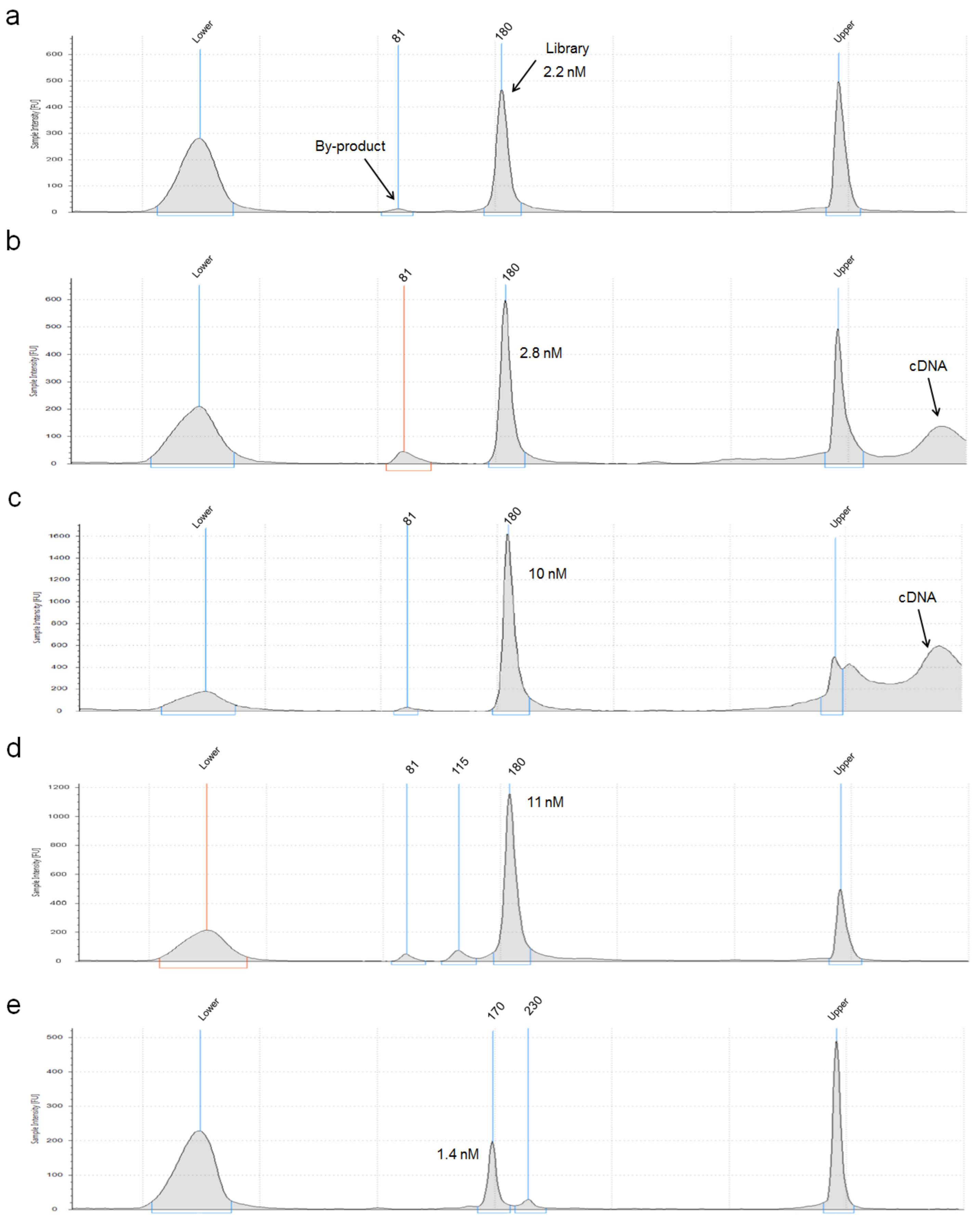

Supplementary Fig. 1. PCR amplified library quality control on gel. Each TapeStation D1000 High Sensitivity (Agilent) electropherogram represents a prepared and sequenced TAC-seq library. The libraries were created for (a) ERCC spike-in assay (180 bp), (b) high sequencing coverage mRNA assay for endometrial receptivity mRNA biomarkers (180 bp), (c) low sequencing coverage mRNA assay for endometrial receptivity mRNA biomarkers (180 bp), (d) cell-free DNA-based assay to detect chromosome copy-number, and (e) 49-plex endometrial miRNA assay (170 bp). 81 bp is the expected by-product, having only Illumina P7 common motif and providing no complete clusters on sequencing flow-cell. Low peak at 115 bp is a by-product generated by two PCR primers in a combination of right detector probe matching UMI motif and simultaneous contribution from specific part. The 115 bp by-product generates clusters on flow-cell and provides a read starting with the motif GGAGCTGTCTGCGACTTT(BARCODE). 230 bp band in the miRNA assay is a by-product with unknown origin. As TAC-seq is a single-tube assay, cDNA (>1500 bp) is carried from reverse transcriptase to final QC. cDNA does not affect sequencing outcome but is visible on both mRNA assays (b and c) and is easily removable by an additional bead-based purification step. The cDNA mass may affect library quantification. Here-presented library concentrations were measured by qPCR-based assay without influence from cDNA.

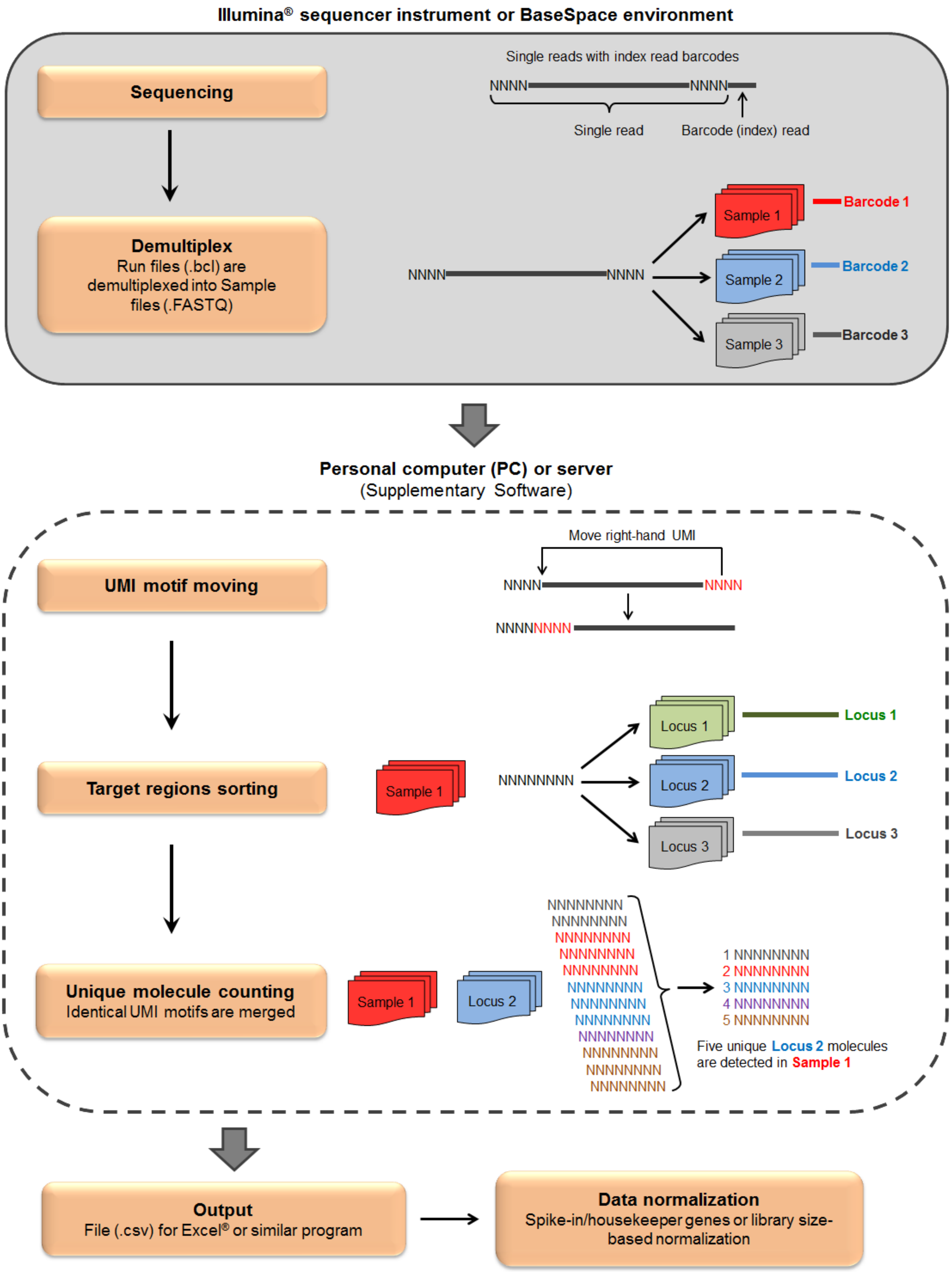

Supplementary Fig. 2. Overview of data analysis. Sequencing data are first quality filtered and sorted (demultiplexed) by Illumina® software based on Barcode (index) reads between analysed Samples (grey box). UMI motifs are joined, target regions are sorted between loci and PCR effect is reduced by merging of identical UMI motifs (dashed box). As TAC-seq data analysis does not need sequencing read mapping or similar resource-demanding computing, the manipulations can be performed in personal computer (PC) using open-source software from **https://github.com/cchtEE/TAC-seq-data-analysis**

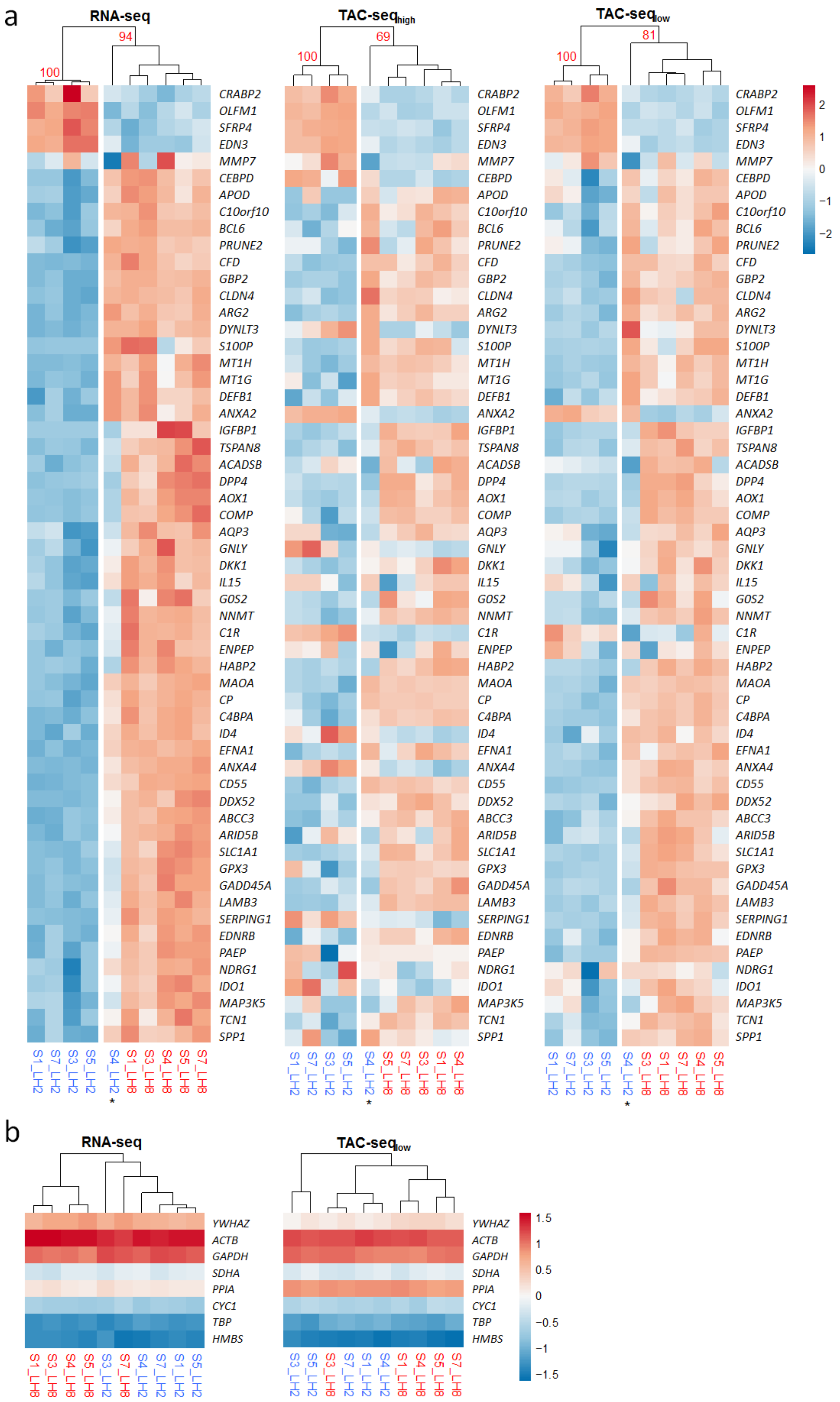

Supplementary Fig. 3. Clustering analysis of biomarker genes and housekeepers through RNA-seq and TAC-seq approaches. Clustering comparison of (a) 57 biomarker genes between full transcriptome RNA-seq (left), high sequencing coverage TAC-seq (middle) and low sequencing coverage TAC-seq (right). Five pre-receptive (blue, LH2 - luteinizing hormone peak detection time plus two days) human uterine endometrial samples were analysed together with five receptive endometrial (red, LH8 - LH peak detection time plus eight days) samples. The one pre-receptive sample (indicated with an asterisk) clusters together with receptive samples through all three comparisons. The data, analysed at UMI threshold 2 (UMI=2) is plotted as row-wise scaled log-transformed CPM values. The samples are hierarchically clustered column-wise using Pearson correlation and clustering probabilities are marked with red numbers. The genes are ordered row-wise by RNA-seq clustering results using Euclidean distance. (b) Heatmap clustering of eight housekeeping genes between RNA-seq (left) and low sequencing coverage TAC-seq (right) demonstrates non-fluctuating gene expression between pre-receptive and receptive samples.

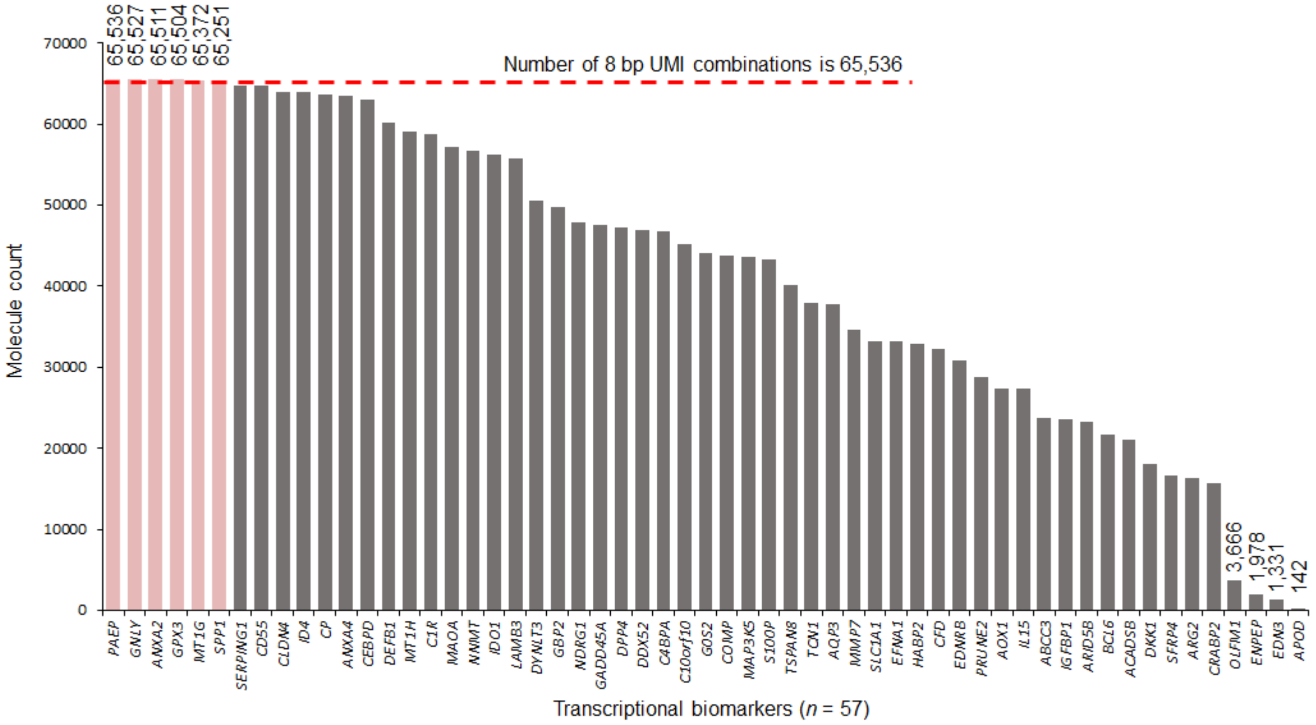

Supplementary Fig. 4. Unique molecule counts and UMI induced saturation. Molecule read count (Y-axis) of studied biomarker set after UMI correction. All analysed 57 transcripts (X-axis) were detected by sequencing and top six highly expressed genes were facing UMI-length restricted 'technical' saturation. Red dashed line presents the possible number of combinations (65,536) in case of 8-bp UMI.

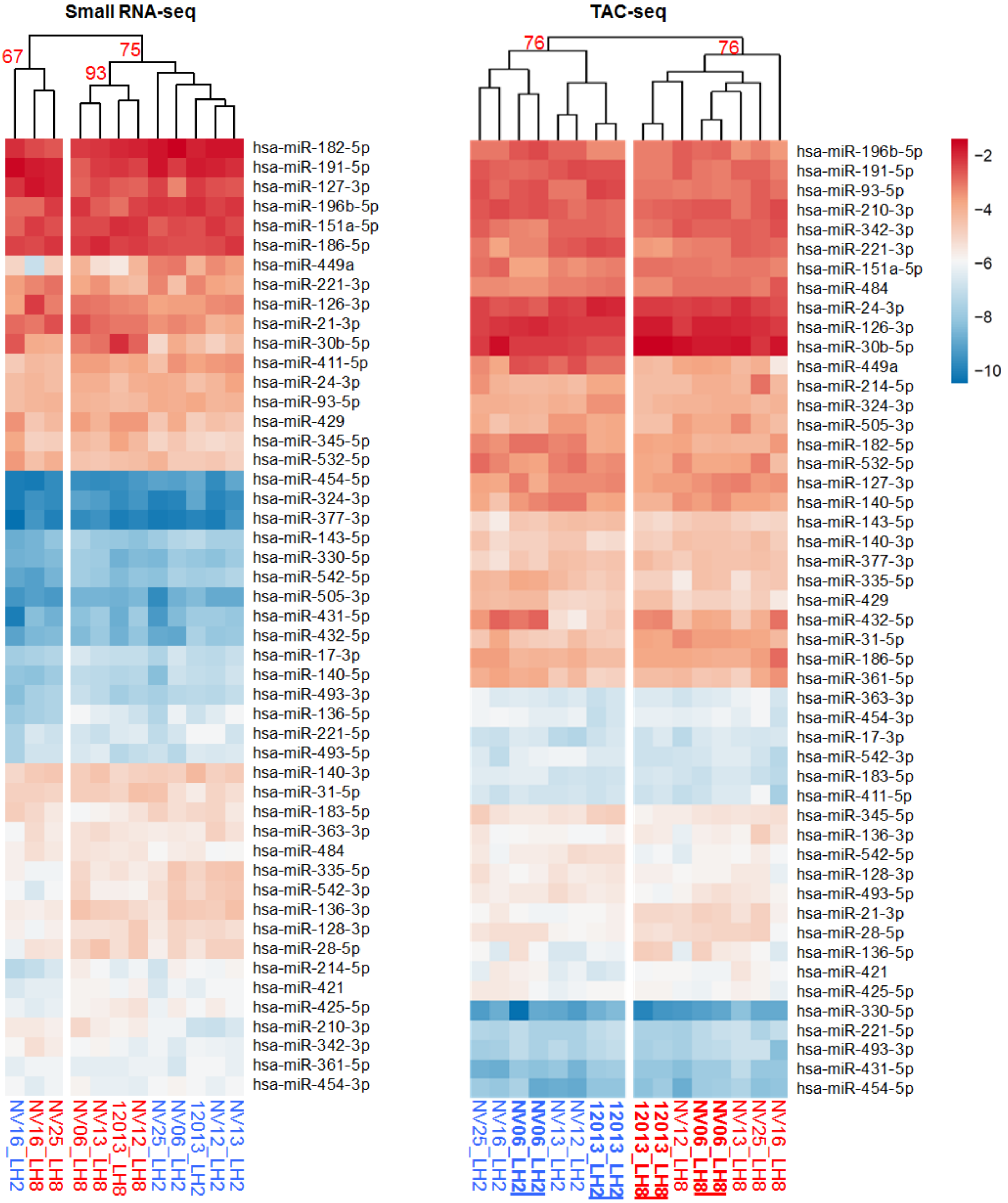

Supplementary Fig. 5. Clustering of miRNA profiles. Heatmaps of the small RNA-seq (left) and targeted miRNA TAC-seq (right). TAC-seq assay demonstrates the sensitivity to distinguish endometrial samples at pre-receptive (blue, LH2) and receptive (red, LH8) time points of the cycle. In total, two biopsies (LH2 and LH8) from six individuals, altogether 12 samples were analysed. Four samples were performed as technical replicates (in bold and underlined) in TAC-seq assay.

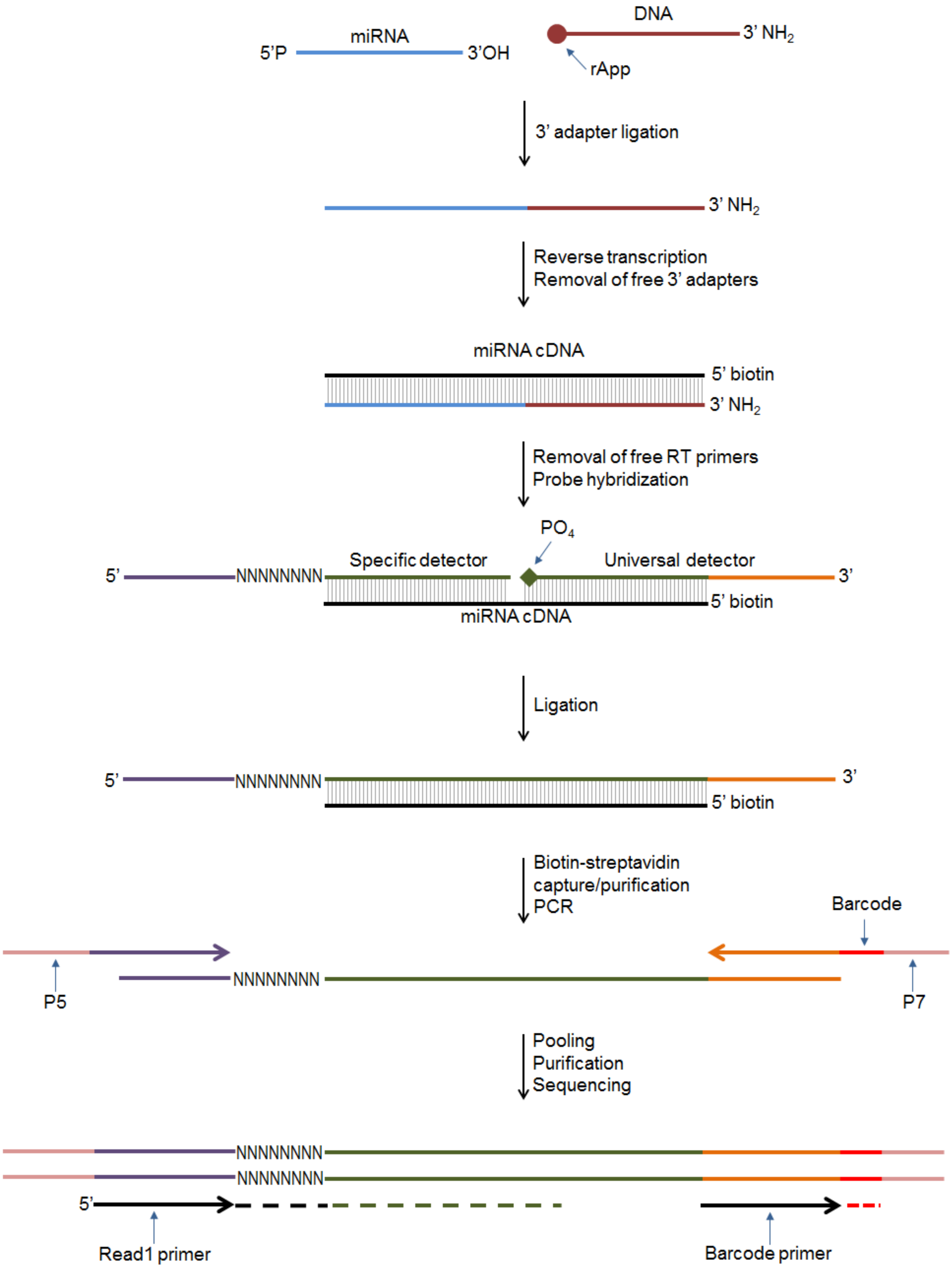

Supplementary Fig. 6. Schematic outline of miRNA TAC-seq library preparation. Total-RNA is introduced for 3' adapter ligation. Free adapters were removed enzymatically and cDNA synthesis was initiated by 5' biotinylated primer. Specific TAC-seq detector oligonucleotide hybridizing under stringent conditions on studied miRNA cDNA. Specific detector oligonucleotide has a 20-24 bp specific region in 3' end, eight base pair unique molecular identifier (UMI) motif and a common sequence (purple). The universal TAC-seq detector is 5' phosphorylated and supplied with a common sequence (orange). After thermostable ligation, the biotin enriched cDNA-detectors complex is captured by streptavidin magnetic beads. PCR is used to introduce individual barcodes and Illumina technology-compatible motifs like P5, P7 and complete H10 sequence for Read1 primer. Samples are pooled, purified and concentrated. The created library has a 170 nucleotide amplicon as shown in Supplementary Fig. 1e. A single 32 bp sequencing read is sufficient to analyse 8 bp UMI (black dashed line) and 24 bp miRNA sequence (green dashed line). Barcode is sequenced by independent custom LNA primer (Supplementary Table 3).

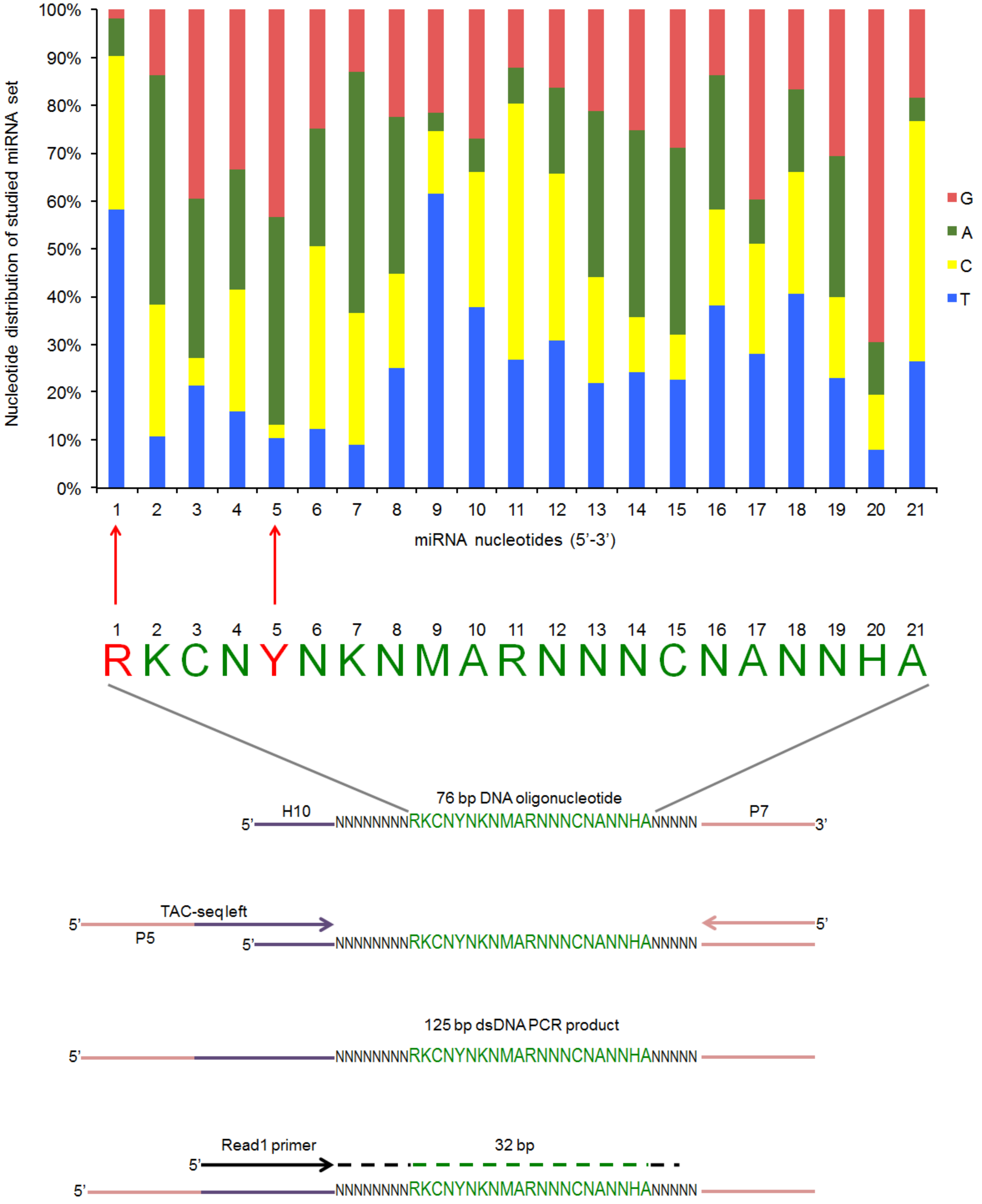

Supplementary Fig. 7. Pooled miRNA nucleotide distribution and the principle of miRNA specific spike-in. miRNA nucleotide distribution of this specific 49-plex set was calculated based on previous small RNA-seq data. Significant unbalance was predicted at positions one and five, and moderate unbalance over multiple sites. Green spike-in DNA sequence motif was designed to compensate low-presented nucleotides during sequencing. The specific green sequence was enriched with left universal sequence, eight-nucleotide UMI, four-nucleotide random nucleotides at right hand and another right-side universal sequence. The synthetic 76 bp DNA oligonucleotide was used as a template of low-cycle PCR, purified and quantified to 2 nM spike-in solution. The full sequence of „miRNA spike-in" DNA oligonucleotide is in Supplementary Table 3 and its preparation is described in Supplementary Methods.

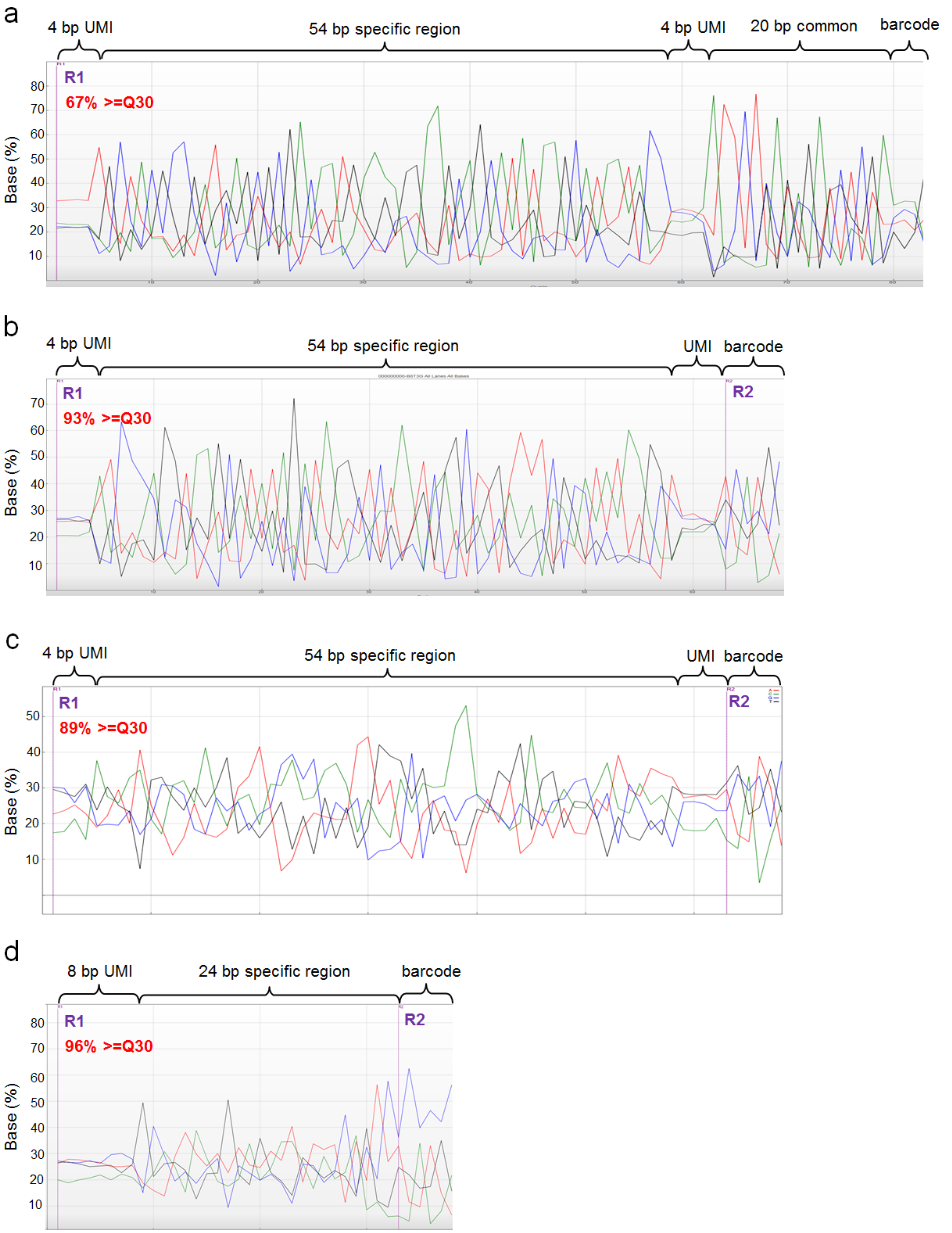

Supplementary Fig. 8. Library complexity through different TAC-seq assays. The plots show the percentage of clusters of which each base has been called through Read1 (R1) and barcode read (R2). (a) Endometrial mRNA library was sequenced with high-coverage using a long single-read, calling a 20 bp common motif that together with unbalanced Read1 caused the drop of Q30 score (67%). (b) Low sequencing coverage endometrium biomarker assay was sequenced using a custom LNA barcode primer that avoids the 20 bp common motif (it anneals on it) and increased the Q30 score to 93%. (c) Cell-free DNA trisomy 21 library was also sequenced with 62 bp single read and following 6 bp barcode primed by custom LNA barcode primer. Sequencing protocol with 62 bp Read1 and following custom barcode primer is optimal to ensure best quality reads. (d) miRNA reads started with eight UMI nucleotides and continued with a 24 bp specific region in Read1, followed by 6 barcode nucleotides by custom LNA barcode primer.

a

	Trisomy factor (%)							
	**0**	**5**	**10**	**15**	**20**	**25**	**30**	**100**
Z	-4.2	4.8	43	13.2	18.2	22.7	24.4	62.1
ZZ	-0.3	0.4	4.2	1.2	1.8	2.2	2.6	4.6
BM	-2.9	-1.2	8	1.2	2.9	3.1	4.4	12.4
OM	6.1	5.4	1.7	4.6	4.3	4	3.5	0.8
PCR product concentration*	13.1	12	9	8.9	12.5	9.8	9.8	9.2
Ch2 reads	991,648	835,726	4,063,932	1,046,881	870,644	922,930	1,081,705	1,595,939
Ch21 reads	213,037	181,753	1,006,146	234,883	195,814	211,642	251,082	406,915
Ch2 average coverage**	0.348	0.293	1.426	0.367	0.306	0.324	0.380	0.560
Ch21 average coverage**	0.388	0.331	1.831	0.427	0.356	0.385	0.457	0.740

* Purified by AMPure XP beads and quantified by Qubit
** 85 bp single-end reads
ZZ score is the standard score of the Z-score of a given autosome in comparison with the Z-scores of remaining autosome.
BM (bin median) is calculated from the median of Z-scores measured per 5 MB bin in the autosome of interest
OM (other median) is the median of the absolute value of the 5 Mb Z-scores over the remaining bins

b

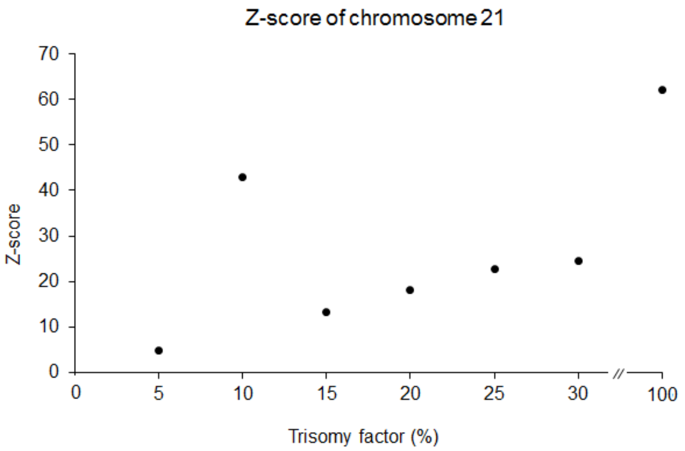

c

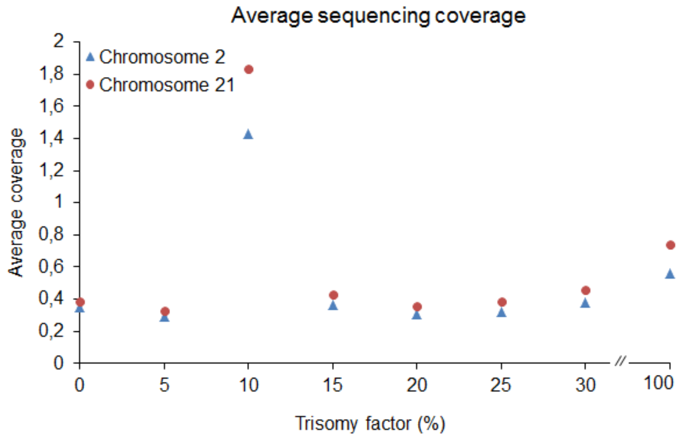

Supplementary Fig. 9. Low coverage genome re-sequencing to detect chromosome 21 trisomy. The table (a) concludes sequencing and data analysis outcomes over eight different chromosome 21 proportions. The same acoustically sheared cell line genomic DNAs were used as in TAC-seq experiment. Factor 0% corresponds to euploid chromosome 21 and 100% to full trisomy 21, respectively. Z-score based trisomy detection indicates aneuploidy already at 5% level (Z-score 4.8) and has an increasing trend to 100%. The 10% sample is interpreted as an outlier due to (b) abnormal Z-score value and (c) significantly higher sequencing coverage compared to the rest of the parallel studied samples.

a

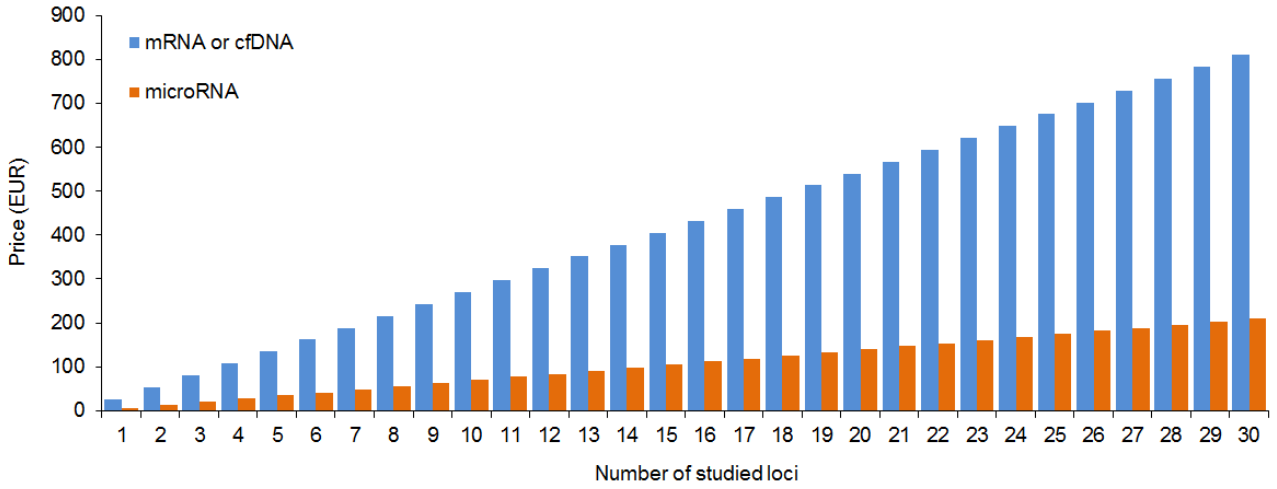

b

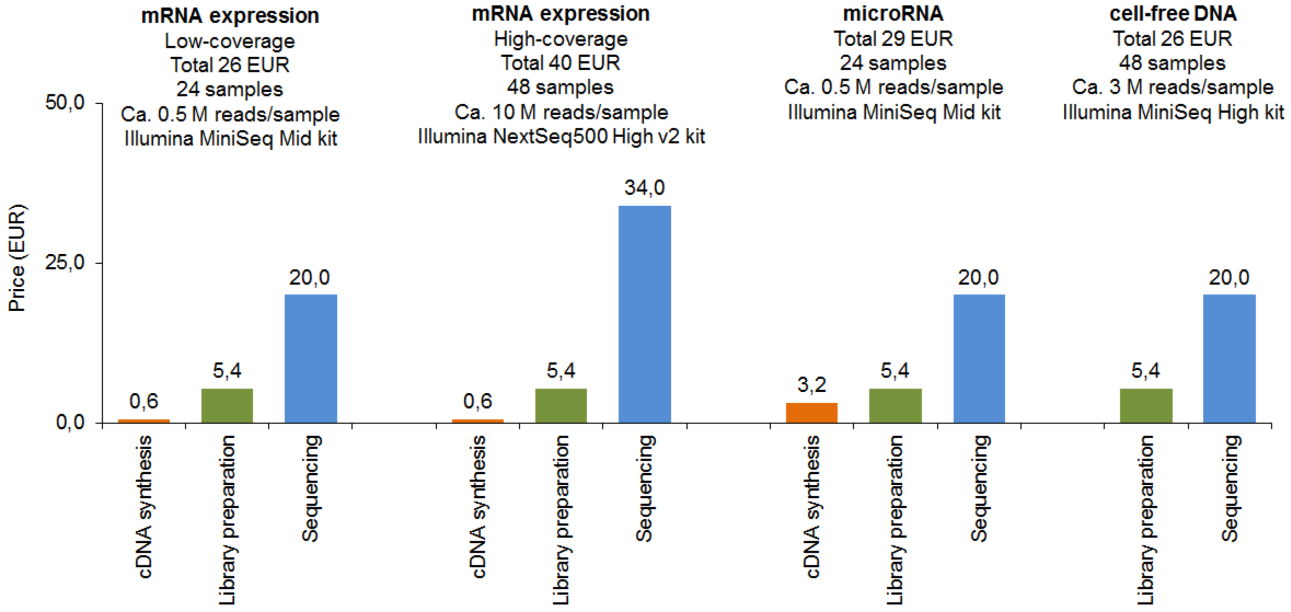

Supplementary Fig. 10. Estimated setup and running cost of different TAC-seq applications. Setup cost of TAC-seq depends on number of studied loci due to the need of specific detector oligonucleotides. (a) mRNA and cell-free DNA loci need two specific detector oligonucleotides where right hand is 5' phosphorylated. As miRNA assay uses only one specific unmodified detector oligonucleotide and an universal phosphorylated detector oligonucleotide for all loci, the setup cost is significantly lower compared to mRNA and cell-free DNA. (b) Reagent costs are provided per each application highlighting the rough estimation of cDNA synthesis, library preparation and estimated sequencing costs based on required consumables and sequencing depth (see in Supplementary Table 2).

Supplementary Table 1.

ERCC spike-in molecule calculation and sequencing outcome at different UMI thresholds

Assay ID	ERCC ID	ERCC group	mRNA length (bp)	Concentration in Mix 1 (attomoles/µl). Based on ERCC manual	Molecules in Mix 1 (molecules/µl)	100× dilution (molecules/µl). Standard storage aliquot. Diluted with water	Molecules in 1.3 µl storage aliquot	Dilution prior reverse transcriptase. Add 199 µl water (extra 153× dilution). 15300×	Molecules added to RT master mix	Assayd molecules	Average raw reads per replicate* (UMI = 0)
1	ERCC-00130	A	1037	30000,00	18066424500,00	180664245,00	234863518,50	1180218,69	1180218,69	Not designed	NA
2	ERCC-00004	A	499	7500,00	4516606125,00	45166061,25	58715879,63	295054,67	295054,67	+	21361456
3	ERCC-00136	A	1011	1875,00	1129151531,25	11291515,31	14678969,91	73763,67	73763,67	+	7879306
4	ERCC-00108	A	997	937,50	564575765,63	5645757,66	7339484,95	36881,83	36881,83	+	2406283
5	ERCC-00116	A	1969	468,75	282287882,81	2822878,83	3669742,48	18440,92	18440,92	+	485700
6	ERCC-00092	A	1100	234,38	141143941,41	1411439,41	1834871,24	9220,46	9220,46	+	436326
7	ERCC-00095	A	499	117,19	70571970,70	705719,71	917435,62	4610,23	4610,23	+	606145
8	ERCC-00131	A	747	117,19	70571970,70	705719,71	917435,62	4610,23	4610,23	+	10915
9	ERCC-00062	A	999	58,59	35285985,35	352859,85	458717,81	2305,11	2305,11	+	122368
10	ERCC-00019	A	619	29,30	17642992,68	176429,93	229358,90	1152,56	1152,56	+	14704
11	ERCC-00144	A	513	29,30	17642992,68	176429,93	229358,90	1152,56	1152,56	+	6082
12	ERCC-00170	A	999	14,65	8821496,34	88214,96	114679,45	576,28	576,28	+	45843
13	ERCC-00154	A	513	7,32	4410748,17	44107,48	57339,73	288,14	288,14	+	8327
14	ERCC-00085	A	820	7,32	4410748,17	44107,48	57339,73	288,14	288,14	+	7622
15	ERCC-00028	A	1106	3,66	2205374,09	22053,74	28669,86	144,07	144,07	+	4514
16	ERCC-00033	A	2000	1,83	1102687,04	11026,87	14334,93	72,03	72,03	+	1704
17	ERCC-00134	A	249	1,83	1102687,04	11026,87	14334,93	72,03	72,03	+	1380
18	ERCC-00147	A	999	0,92	551343,52	5513,44	7167,47	36,02	36,02	+	1640
19	ERCC-00097	A	498	0,46	275671,76	2756,72	3583,73	18,01	18,01	+	211
20	ERCC-00156	A	470	0,46	275671,76	2756,72	3583,73	18,01	18,01	+	474
21	ERCC-00123	A	998	0,23	137835,88	1378,36	1791,87	9,00	9,00	+	181
22	ERCC-00017	A	1113	0,11	68917,94	689,18	895,93	4,50	4,50	+	268
23	ERCC-00083	A	999	0,03	17229,49	172,29	223,98	1,13	1,13	+	500

* Average calculation over seven replicates

Supplementary Table 1 continues

UMI corrected average read count per replicate (UMI = 1)	UMI corrected average read count per replicate (UMI = 2)	UMI corrected average read count per replicate (UMI = 3)	UMI corrected average read count per replicate (UMI = 4)	UMI corrected average read count per replicate (UMI = 5)	UMI corrected average read count per replicate (UMI = 6)	UMI corrected average read count per replicate (UMI = 7)	UMI corrected average read count per replicate (UMI = 8)	UMI corrected average read count per replicate (UMI = 9)	UMI corrected average read count per replicate (UMI = 10)
NA	NA	NA	NA	NA	NA	NA	NA	NA	NA
64007	61789	59465	57324	55435	53800	52420	51216	50217	49346
59299	50973	43239	36942	32162	28625	26025	24179	22850	21915
41960	27760	20187	16301	14296	13250	12690	12397	12236	12143
19783	8368	5425	4593	4342	4248	4207	4184	4168	4156
11488	5202	4039	3753	3666	3635	3617	3607	3597	3591
13228	5932	3991	3344	3115	3016	2976	2956	2943	2934
2249	1485	1371	1248	1093	917	732	564	423	292
3684	1473	1127	1047	1024	1016	1012	1007	1005	1003
846	456	427	421	420	418	417	415	414	412
678	457	445	437	429	417	400	383	356	330
1370	531	418	398	394	392	390	389	388	388
394	126	103	101	100	100	99	99	99	99
401	143	119	115	114	113	113	113	113	113
192	67	55	52	52	51	51	51	50	50
73	34	30	30	30	30	30	30	30	30
57	24	22	21	21	21	21	21	21	21
61	21	16	15	15	15	15	15	15	15
23	15	14	14	14	13	13	12	12	11
23	8	7	7	7	7	7	7	7	7
16	10	9	9	9	9	9	9	8	8
8	3	3	3	2	2	2	2	2	2
11	5	4	4	4	4	4	4	4	4

Supplementary Table 1 continues

Average PCR redundancy per replicate (UMI = 1)	Average PCR redundancy per replicate (UMI = 2)	Average PCR redundancy per replicate (UMI = 3)	Average PCR redundancy per replicate (UMI = 4)	Average PCR redundancy per replicate (UMI = 5)	Average PCR redundancy per replicate (UMI = 6)	Average PCR redundancy per replicate (UMI = 7)	Average PCR redundancy per replicate (UMI = 8)	Average PCR redundancy per replicate (UMI = 9)	Average PCR redundancy per replicate (UMI = 10)		
NA	NA	NA	NA	NA	NA	NA	NA	NA	NA	NA	NA
334	346	359	373	385	397	407	417	425	433	N_A	6,02214E+23
133	154	182	213	245	276	303	326	345	360		
57	87	120	148	169	182	190	194	197	198		
25	58	90	106	112	115	116	116	117	117		
38	84	108	116	119	120	121	121	121	122		
46	102	152	182	195	201	204	205	206	207		
5	7	8	9	10	12	15	19	26	38		
33	83	109	117	120	120	121	121	122	122		
17	32	35	35	35	35	35	35	36	36		
9	13	14	14	14	15	15	16	17	18		
34	86	109	115	116	117	117	118	118	118		
21	66	81	83	83	84	84	84	84	84		
19	53	64	66	67	67	68	68	68	68		
23	67	82	86	87	88	88	89	89	89		
23	50	57	57	57	57	57	57	57	57		
24	57	64	66	66	66	67	67	67	67		
26	78	104	107	109	111	111	112	112	112		
9	15	15	15	16	16	17	18	19	20		
21	60	71	73	73	73	73	73	73	73		
12	19	21	21	21	21	21	21	22	23		
36	94	107	109	114	114	114	114	114	114		
48	104	134	134	134	136	136	136	136	136		

Supplementary Table 2

Reagent cost for experiments used in this study (cDNA synthesis + ligation + PCR + purification and QC)

Reagents	Supplier	Cat #	No. rxn	Price per kit (EUR)	Unitary cost (EUR)
Maxima H Minus Reverse Transcriptase	ThermoFisher	EP0753	2000	522	0,261
dNTP mix	ThermoFisher	R0181	50000	105	0,002
Oligo-T30	SigmaAldrich		10000	10	0,001
RNase inhibitor	ThermoFisher	EO0384	15000	840	0,056
Betaine (5 M solution)	SigmaAldrich	B0300-5VL	5000	115	0,023
Dynabeads MyOne Carboxylic Acid beads	ThermoFisher	65012	2500	512	0,205
T4 RNA Ligase 2, truncated	NewEngland Biolabs	M0242L	250	268	1,072
5’-Deadenylase	NewEngland Biolabs	M0331S	200	68	0,34
Lambda exonuclease	NewEngland Biolabs	M0262L	2000	268	0,134
Exonucease I	ThermoFisher	EN0582	1000	305	0,305
Micro RT biotin primer	SigmaAldrich		1000	10	0,01
RNase inhibitor	ThermoFisher	EO0384	7500	840	0,112
dNTP mix	ThermoFisher	R0181	50000	105	0,002
Maxima H Minus Reverse Transcriptase	ThermoFisher	EP0753	1000	522	0,522
Dynabeads MyOne Streptavidin C1 beads	ThermoFisher	65001	660	463	0,702
Taq DNA ligase	NewEngland Biolabs	M0208L	10000	322	0,032
TAC-seq Left primer	SigmaAldrich		2000	10	0,005
HOT FIREPol Blend Master Mix	SolisBiodyne	04-27-00125	5000	460	0,092
NucleoSpin Gel and PCR Clean-up	Macherey-Nagel	740609.250	250	300	1,200
AMPure XP beads	Beckman Coulter	A63881	1200	1071	0,893
TapeStation High Sensitivity D1000 ScreenTape	Agilent Technologies	5067-5584	120	380	3,167
		**mRNA cDNA synthesis**			**0,55**
		**microRNA cDNA synthesis**			**3,20**
		**ligation/PCR/purification**			**5,39**
		**mRNA sample/library total**			**5,94**
		**microRNA sample/library total**			**8,59**
		**cell-free DNA sample/library total**			**5,39**

Supplementary Table 3

Used oligonucleotides

Name of oligonucleotide	Modification		Sequence (5'-3')	Producer	Purifi-cation
	5'	3'			
Oligo-T30			TTTTTTTTTTTTTTTTTTTTTTTTTTTTTT	Sigma	HPLC
Micro RT biotin	Biotin		GCTCCAGAGACGTGTGCTCTTCCGATCT	Metabion	Desalted
Adenylated 3’ linker	Adenylate	Amine	AGATCGGAAGAGCACACGTCT	NEB	HPLC
TAC-seq left			AATGATACGGCGACCACCGAGATCTACACTAACAACACTCTTTCCCTACAC-GACGCTCTTCCGATCT	Sigma	HPLC
miRNA spike-in			ACGACGCTCTTCCGATCTNNNNNNNNRKCNYNKNMARNNNCNANNHA-NNNNNATCTCGTATGCCGTCTTCTGCTTG	Metabion	Desalted
miRNA spike-in right primer			TAGAGCATACGGCAGAAGACGAAC	Metabion	Desalted
Barcode-seq primer LNA			CT+GGAGCT+GTCTGC+GACTTT	Exiqon	HPLC
TAC-seq barcode 1			CAAGCAGAAGACGGCATACGAGATGATCTGAAAGTCGCAGACAGCTCCAG	Sigma	HPLC
TAC-seq barcode 2			CAAGCAGAAGACGGCATACGAGATGCCTAAAAAGTCGCAGACAGCTCCAG	Sigma	HPLC
TAC-seq barcode 3			CAAGCAGAAGACGGCATACGAGATCGTGATAAAGTCGCAGACAGCTCCAG	Sigma	HPLC
TAC-seq barcode 4			CAAGCAGAAGACGGCATACGAGATTGGTCAAAAGTCGCAGACAGCTCCAG	Sigma	HPLC
TAC-seq barcode 5			CAAGCAGAAGACGGCATACGAGATATTGGCAAAGTCGCAGACAGCTCCAG	Sigma	HPLC
TAC-seq barcode 6			CAAGCAGAAGACGGCATACGAGATCTGATCAAAGTCGCAGACAGCTCCAG	Sigma	HPLC
TAC-seq barcode 7			CAAGCAGAAGACGGCATACGAGATGTAGCCAAAGTCGCAGACAGCTCCAG	Sigma	HPLC
TAC-seq barcode 8			CAAGCAGAAGACGGCATACGAGATTACAAGAAAGTCGCAGACAGCTCCAG	Sigma	HPLC
TAC-seq barcode 9			CAAGCAGAAGACGGCATACGAGATATCAGTAAAGTCGCAGACAGCTCCAG	Sigma	HPLC
TAC-seq barcode 10			CAAGCAGAAGACGGCATACGAGATAGGAATAAAGTCGCAGACAGCTCCAG	Sigma	HPLC
TAC-seq barcode 11			CAAGCAGAAGACGGCATACGAGATTAGTTGAAAGTCGCAGACAGCTCCAG	Sigma	HPLC
TAC-seq barcode 12			CAAGCAGAAGACGGCATACGAGATATCGTGAAAGTCGCAGACAGCTCCAG	Sigma	HPLC
TAC-seq barcode 13			CAAGCAGAAGACGGCATACGAGATTGAGTGAAAGTCGCAGACAGCTCCAG	Sigma	HPLC
TAC-seq barcode 14			CAAGCAGAAGACGGCATACGAGATGCCATGAAAGTCGCAGACAGCTCCAG	Sigma	HPLC
TAC-seq barcode 15			CAAGCAGAAGACGGCATACGAGATTGTTGGAAAGTCGCAGACAGCTCCAG	Sigma	HPLC
TAC-seq barcode 16			CAAGCAGAAGACGGCATACGAGATAGCATCAAAGTCGCAGACAGCTCCAG	Sigma	HPLC
ERCC-00004_L			ACACGACGCTCTTCCGATCTNNNNCCCAATATCAGACATTCCTGTAGATAA	Metabion	Desalted
ERCC-00017_L			ACACGACGCTCTTCCGATCTNNNNCTAGGCGGTTGCGCAAGTAACTTCATC	Metabion	Desalted
ERCC-00019_L			ACACGACGCTCTTCCGATCTNNNNAGGGAGTACGAGCAGTGCACCGTTGAA	Metabion	Desalted
ERCC-00028_L			ACACGACGCTCTTCCGATCTNNNNGGTAAACAACGGGGAATATAATTCAGT	Metabion	Desalted
ERCC-00033_L			ACACGACGCTCTTCCGATCTNNNNAGGTTCCATCACCAAACTCTGGTTATA	Metabion	Desalted
ERCC-00062_L			ACACGACGCTCTTCCGATCTNNNNTCTATGTCTTGCAAAAACGGCTATTGA	Metabion	Desalted
ERCC-00083_L			ACACGACGCTCTTCCGATCTNNNNCACAGTGTCTTTTTTCTTCGTCTAATG	Metabion	Desalted
ERCC-00085_L			ACACGACGCTCTTCCGATCTNNNNTCAACAAGGGTAATCCCTCCGACAACC	Metabion	Desalted
ERCC-00092_L			ACACGACGCTCTTCCGATCTNNNNGCGTTTTTTTGTCGTTGTCGCAGAACG	Metabion	Desalted
ERCC-00095_L			ACACGACGCTCTTCCGATCTNNNNTTGGGCCAAATGCAACATTATCATAGA	Metabion	Desalted
ERCC-00097_L			ACACGACGCTCTTCCGATCTNNNNCTAATTCCAACAGTTTCAGCCAACAAA	Metabion	Desalted

ERCC-00108_L			ACACGACGCTCTTCCGATCTNNNNGACTGTGCGCTCATAGCCGACACTGTG	Metabion	Desalted
ERCC-00116_L			ACACGACGCTCTTCCGATCTNNNNCTGAGACACTGATCGAGCATTAAGACT	Metabion	Desalted
ERCC-00123_L			ACACGACGCTCTTCCGATCTNNNNCCAGTACCTCCTTTTCCAGATGCTATC	Metabion	Desalted
ERCC-00130_L			ACACGACGCTCTTCCGATCTNNNNTAAAGAAGCGATTCAGCGCTATTTGCG	Metabion	Desalted
ERCC-00131_L			ACACGACGCTCTTCCGATCTNNNNCTAGTATTGGCTCCTGTCCACATGGTC	Metabion	Desalted
ERCC-00134_L			ACACGACGCTCTTCCGATCTNNNNCGCTCGTTCAATAGATTTAGTAACTAC	Metabion	Desalted
ERCC-00136_L			ACACGACGCTCTTCCGATCTNNNNACTTCGCAAAGACGATTGACTAGTTTC	Metabion	Desalted
ERCC-00144_L			ACACGACGCTCTTCCGATCTNNNNGGCACATAATCAAGTCTACATCAATCA	Metabion	Desalted
ERCC-00147_L			ACACGACGCTCTTCCGATCTNNNNGAAGCTCCAGGTATTCCACCAGCTAAG	Metabion	Desalted
ERCC-00154_L			ACACGACGCTCTTCCGATCTNNNNAGTCCACGAGTTACAGCCAGCGGGTTT	Metabion	Desalted
ERCC-00156_L			ACACGACGCTCTTCCGATCTNNNNGACTAGTCGAATCTTAGGGTTGTATGC	Metabion	Desalted
ERCC-00170_L			ACACGACGCTCTTCCGATCTNNNNCTGTGTTCCAGCTACAAACTTAGAAAC	Metabion	Desalted
ERCC-00004_R	Pho		AATCACCGGCTTGCCTGTTTTTGCCACNNNNCTGGAGCTGTCTGCGACTTT	Metabion	Desalted
ERCC-00017_R	Pho		ATGTATCGCTGGGGAATAATGTTCCTGNNNNCTGGAGCTGTCTGCGACTTT	Metabion	Desalted
ERCC-00019_R	Pho		ACAAGCACAGGAGGTATGAAGCATCAGNNNNCTGGAGCTGTCTGCGACTTT	Metabion	Desalted
ERCC-00028_R	Pho		TGAACCGGTGTGGAGCCTGCACTTGGANNNNCTGGAGCTGTCTGCGACTTT	Metabion	Desalted
ERCC-00033_R	Pho		CAATGGCTACATTGGCAAATGCATTAANNNNCTGGAGCTGTCTGCGACTTT	Metabion	Desalted
ERCC-00062_R	Pho		AGCAATCCTCTCCCCAATACTTAAAAANNNNCTGGAGCTGTCTGCGACTTT	Metabion	Desalted
ERCC-00083_R	Pho		TTTCATAGCCTTCTGGAATTTCTTCCTNNNNCTGGAGCTGTCTGCGACTTT	Metabion	Desalted
ERCC-00085_R	Pho		CTCAGTGTTATCATCCGCGTCAAGGGGNNNNCTGGAGCTGTCTGCGACTTT	Metabion	Desalted
ERCC-00092_R	Pho		CGATTTGCTCCGAAAGCTTTAAGCCGTNNNNCTGGAGCTGTCTGCGACTTT	Metabion	Desalted
ERCC-00095_R	Pho		TGCTCATAGCAAAAGGATTTGGTTTTGNNNNCTGGAGCTGTCTGCGACTTT	Metabion	Desalted
ERCC-00097_R	Pho		CCAATAGCATCAAACCCATGTCATGGCNNNNCTGGAGCTGTCTGCGACTTT	Metabion	Desalted
ERCC-00108_R	Pho		CTCGATAAGACCACGCTGTGCGGATATNNNNCTGGAGCTGTCTGCGACTTT	Metabion	Desalted
ERCC-00116_R	Pho		CTAGAGCGGCCGCCGACTAGTGAGCTCNNNNCTGGAGCTGTCTGCGACTTT	Metabion	Desalted
ERCC-00123_R	Pho		GCGATAGCTATTCCATTAATGTCACCANNNNCTGGAGCTGTCTGCGACTTT	Metabion	Desalted
ERCC-00130_R	Pho		CCAAGAACTGTTAACGTCTTGAATTCTNNNNCTGGAGCTGTCTGCGACTTT	Metabion	Desalted
ERCC-00131_R	Pho		GGGTTTTCCGCCCCCAAACATGCAAACNNNNCTGGAGCTGTCTGCGACTTT	Metabion	Desalted
ERCC-00134_R	Pho		TGCCTTACAAATAGCTACTGAGATGCTNNNNCTGGAGCTGTCTGCGACTTT	Metabion	Desalted
ERCC-00136_R	Pho		CCTTGTGAACTAGGATTTTCCCGGGTANNNNCTGGAGCTGTCTGCGACTTT	Metabion	Desalted
ERCC-00144_R	Pho		TGAATGGTTTCTGATTTGCTACCATCANNNNCTGGAGCTGTCTGCGACTTT	Metabion	Desalted
ERCC-00147_R	Pho		CACAGAAGTGGAAGACATTAAAAACCTNNNNCTGGAGCTGTCTGCGACTTT	Metabion	Desalted
ERCC-00154_R	Pho		TAAGGGGGTATTAGCATCTCGAGTGAGNNNNCTGGAGCTGTCTGCGACTTT	Metabion	Desalted
ERCC-00156_R	Pho		TAGAACGGCATGGTATAAGCCGTGCTCNNNNCTGGAGCTGTCTGCGACTTT	Metabion	Desalted
ERCC-00170_R	Pho		AAGTGGAGCTGAGATTACAGCAGAGAANNNNCTGGAGCTGTCTGCGACTTT	Metabion	Desalted
PPIA-1638_L			ACACGACGCTCTTCCGATCTNNNNGACTTGTGTTTTATCTTAACCACCAGA	Metabion	Desalted
CYC1-83_L			ACACGACGCTCTTCCGATCTNNNNGCCATCCCAGGCCTGTTCAGGCCTCAG	Metabion	Desalted
YWHAZ-4145_L			ACACGACGCTCTTCCGATCTNNNNGCTGAAGCAGGAGAAGGAGGGGAAAAT	Metabion	Desalted
GAPDH-58_L			ACACGACGCTCTTCCGATCTNNNNACACTGAATCTCCCCTCCTCACAGTTG	Metabion	Desalted
HMBS-36_L			ACACGACGCTCTTCCGATCTNNNNAGTATGTGGGGGCTTCATCTCTTTAGA	Metabion	Desalted
TBP-343_L			ACACGACGCTCTTCCGATCTNNNNCTGTGAGTTGCTCATACCGTGCTGCTA	Metabion	Desalted
ACTB-5_L			ACACGACGCTCTTCCGATCTNNNNCAGGGCTTACCTGTACACTGACTTGAG	Metabion	Desalted
SDHA-333_L			ACACGACGCTCTTCCGATCTNNNNGGACGTTGGCACTGGGAAGGTCACTCT	Metabion	Desalted

CFD-416_L			ACACGACGCTCTTCCGATCTNNNNGCGGCAACCGCAAGAAGCCCGGGATCT	Metabion	Desalted
MT1H-87_L			ACACGACGCTCTTCCGATCTNNNNTGTCGGGACAGCCCTGCTGTCAGATGA	Metabion	Desalted
GADD45A-518_L			ACACGACGCTCTTCCGATCTNNNNACGGTGATGGCATCTGAATGAAAATAA	Metabion	Desalted
MT1G-88_L			ACACGACGCTCTTCCGATCTNNNNACAGCCCTGCTCCCAAGTACAAATAGA	Metabion	Desalted
IL15-984_L			ACACGACGCTCTTCCGATCTNNNNGTTTTTCTGTCAAGAAGATGATCAGAC	Metabion	Desalted
OLFM1-2052_L			ACACGACGCTCTTCCGATCTNNNNGTACGTGGAGAAGATGGAGAACCAAAT	Metabion	Desalted
CEBPD-64_L			ACACGACGCTCTTCCGATCTNNNNGACTTTTCAGACAAACCCTTTGTATTG	Metabion	Desalted
EDN3-1589_L			ACACGACGCTCTTCCGATCTNNNNAGCAAGCAGGCTTTAGACCTCCACCAT	Metabion	Desalted
G0S2-76_L			ACACGACGCTCTTCCGATCTNNNNGTGTGAATTATCTAAATGCGTCTACCA	Metabion	Desalted
GNLY-146_L			ACACGACGCTCTTCCGATCTNNNNCGCTTCCTCGATCCAGAATCCACTCTC	Metabion	Desalted
DEFB1-107_L			ACACGACGCTCTTCCGATCTNNNNTTTACCAAAATTCAAGGCACCTGTTAC	Metabion	Desalted
PAEP-247_L			ACACGACGCTCTTCCGATCTNNNNATGACGTGGTCATCTGTGTCGCCATCC	Metabion	Desalted
IGFBP1-70_L			ACACGACGCTCTTCCGATCTNNNNTTACATAATCAAAGCTACCTGTGGTGA	Metabion	Desalted
DYNLT3-360_L			ACACGACGCTCTTCCGATCTNNNNAGAGAGCGGAACCATAACTCATTGAAT	Metabion	Desalted
CRABP2-160_L			ACACGACGCTCTTCCGATCTNNNNAAGAGCCCAGATCACCCATTCCGGGTT	Metabion	Desalted
NDRG1-1530_L			ACACGACGCTCTTCCGATCTNNNNAAGAGTGAGCTCTGGTGGAGACAAATG	Metabion	Desalted
ID4-39_L			ACACGACGCTCTTCCGATCTNNNNCACTATAGCTATGTTACGCTAAGCTAC	Metabion	Desalted
MMP7-44_L			ACACGACGCTCTTCCGATCTNNNNGTGTGACTGTGTCTTATTCATCTATAC	Metabion	Desalted
ANXA4-1586_L			ACACGACGCTCTTCCGATCTNNNNACATCTGGAGACTACAGGAAAGTACTG	Metabion	Desalted
TSPAN8-99_L			ACACGACGCTCTTCCGATCTNNNNAGCTGTCTTTTTAAAATGTCTCGGCTA	Metabion	Desalted
EDNRB-2862_L			ACACGACGCTCTTCCGATCTNNNNTCCTGCATTAACCCAATTGCTCTGTAT	Metabion	Desalted
NNMT-833_L			ACACGACGCTCTTCCGATCTNNNNCAAAGTTATTCTTCCACCATGGCCAAC	Metabion	Desalted
CLDN4-221_L			ACACGACGCTCTTCCGATCTNNNNTTGCCCAGCTCTGTGGCCTCAGGACTC	Metabion	Desalted
EFNA1-62_L			ACACGACGCTCTTCCGATCTNNNNGCCCACGTGTATAGTATCTGTATATAA	Metabion	Desalted
COMP-138_L			ACACGACGCTCTTCCGATCTNNNNTGACACCATCCCAGAGGACTATGAGAC	Metabion	Desalted
CD55-1548_L			ACACGACGCTCTTCCGATCTNNNNGAAACAACCCCAAATAAAGGAAGTGGA	Metabion	Desalted
DKK1-175_L			ACACGACGCTCTTCCGATCTNNNNTTGTGTGTGTGTACGTATGTGTGTGTT	Metabion	Desalted
SPP1-313_L			ACACGACGCTCTTCCGATCTNNNNTGGCTTCATGGAAACTCCCTGTAAACT	Metabion	Desalted
AQP3-170_L			ACACGACGCTCTTCCGATCTNNNNTAATGCAGGCATGAAGGGTGGAGTGAA	Metabion	Desalted
S100P-29_L			ACACGACGCTCTTCCGATCTNNNNCTTCCCAAAAGTGTTTGTTGGCAATTA	Metabion	Desalted
APOD-94_L			ACACGACGCTCTTCCGATCTNNNNTCCCCTACCCCCCCCCCATAAAGACAA	Metabion	Desalted
ACADSB-4320_L			ACACGACGCTCTTCCGATCTNNNNCTGTTTAACTTAGGCACAGGAGATCCA	Metabion	Desalted
C10orf10-368_L			ACACGACGCTCTTCCGATCTNNNNAGCAAGAAGGTGAGGCATCAGGGAACG	Metabion	Desalted
ABCC3-4217_L			ACACGACGCTCTTCCGATCTNNNNCGCTTTCATGGTCTTGCTGATTCCACT	Metabion	Desalted
TCN1-40_L			ACACGACGCTCTTCCGATCTNNNNTATCCCAGTACGAGCAGGAGAGTTAAT	Metabion	Desalted
IDO1-153_L			ACACGACGCTCTTCCGATCTNNNNCTGTATGCATTCCTGTCATTACCCATT	Metabion	Desalted
GPX3-34_L			ACACGACGCTCTTCCGATCTNNNNTTCGGAGGACGTGCCCTCACCCCTCAC	Metabion	Desalted
BCL6-1192_L			ACACGACGCTCTTCCGATCTNNNNCAGGAGAGAAACCTTACCATTGTGAGA	Metabion	Desalted
ANXA2-239_L			ACACGACGCTCTTCCGATCTNNNNAAGGAGTTGGAAGTGAAGTCTATGATG	Metabion	Desalted
SFRP4-23_L			ACACGACGCTCTTCCGATCTNNNNCAACAAACTGTTGTGCTATTGGATACT	Metabion	Desalted
SERPING1-81_L			ACACGACGCTCTTCCGATCTNNNNGGGTCTGGGCAAGGGACCTGCTTCTAT	Metabion	Desalted
ARG2-34_L			ACACGACGCTCTTCCGATCTNNNNAGCTGTCACTTAGGGATAACACTGTCT	Metabion	Desalted
C1R-156_L			ACACGACGCTCTTCCGATCTNNNNAAGACCGTGTGTGAAATTCTCTTTCCT	Metabion	Desalted

C4BPA-161_L			ACACGACGCTCTTCCGATCTNNNNCCTCTTGCAATTCAATACAGATCAGTT	Metabion	Desalted
GBP2-987_L			ACACGACGCTCTTCCGATCTNNNNCCTCTCCCCAAGAAACAACATGAATGA	Metabion	Desalted
LAMB3-174_L			ACACGACGCTCTTCCGATCTNNNNGCCAATGGGACAGTTACACTTGACAGA	Metabion	Desalted
ARID5B-3946_L			ACACGACGCTCTTCCGATCTNNNNCATTTACCCTTTAGCTGCTATAAATCC	Metabion	Desalted
DPP4-427_L			ACACGACGCTCTTCCGATCTNNNNCTCAGGAAATCAAATATGCAAAGCACT	Metabion	Desalted
SLC1A1-123_L			ACACGACGCTCTTCCGATCTNNNNGTTCTACCCCTTACTAGGTTGCCCCAA	Metabion	Desalted
HABP2-18_L			ACACGACGCTCTTCCGATCTNNNNTTGTTTGAGCTGCGTTTCACACTTCTT	Metabion	Desalted
MAOA-59_L			ACACGACGCTCTTCCGATCTNNNNGTGCTACACGTTGGAGTATACCTATGT	Metabion	Desalted
PRUNE2-190_L			ACACGACGCTCTTCCGATCTNNNNCGTCTTATCACAATGCCTCAGTAGTTT	Metabion	Desalted
DDX52-5_L			ACACGACGCTCTTCCGATCTNNNNGCGAGACTATCAAAGGGCCCTTCAGGA	Metabion	Desalted
CP-45_L			ACACGACGCTCTTCCGATCTNNNNCCTTAAAGTGTTCTTGGGATGAAAATG	Metabion	Desalted
MAP3K5-749_L			ACACGACGCTCTTCCGATCTNNNNACGTGATGACTTAAAATGCTTGAGACT	Metabion	Desalted
ENPEP-314_L			ACACGACGCTCTTCCGATCTNNNNTGGAATAGAACTTAGCCAGCACAGAGT	Metabion	Desalted
AOX1-528_L			ACACGACGCTCTTCCGATCTNNNNGGTGATATCCGTCATTACTCTGTCTCT	Metabion	Desalted
CFD-416_R	Pho		ACACCCGCGTGGCGAGCTATGCGGCCTNNNNCTGGAGCTGTCTGCGACTTT	Metabion	Desalted
MT1H-87_R	Pho		AAACAGAATGACACGTAAAATCCAGGANNNNCTGGAGCTGTCTGCGACTTT	Metabion	Desalted
GADD45A-518_R	Pho		CTGAACCAAATTGCACTGAAGTTTTTGNNNNCTGGAGCTGTCTGCGACTTT	Metabion	Desalted
MT1G-88_R	Pho		GTGACCCGTAAAATCCAGGATTTTTTGNNNNCTGGAGCTGTCTGCGACTTT	Metabion	Desalted
IL15-984_R	Pho		CTTGGATCAGATGAACTCTTAGAAATGNNNNCTGGAGCTGTCTGCGACTTT	Metabion	Desalted
OLFM1-2052_R	Pho		GAAAGGACTGGAGTCCAAGTTCAAACANNNNCTGGAGCTGTCTGCGACTTT	Metabion	Desalted
CEBPD-64_R	Pho		TAGATAAGAGGAAAAGACTGAGCATGCNNNNCTGGAGCTGTCTGCGACTTT	Metabion	Desalted
EDN3-1589_R	Pho		CCAAAGCTCATGCCCGGCAGTGGACTCNNNNCTGGAGCTGTCTGCGACTTT	Metabion	Desalted
G0S2-76_R	Pho		TTTTGCACTAGGGAGGAAGGATAAATGNNNNCTGGAGCTGTCTGCGACTTT	Metabion	Desalted
GNLY-146_R	Pho		CAGTCTCCCTCCCCTGACTCCCTCTGCNNNNCTGGAGCTGTCTGCGACTTT	Metabion	Desalted
DEFB1-107_R	Pho		AGAGGGAAGGCCAAGTGCTGCAAGTGANNNNCTGGAGCTGTCTGCGACTTT	Metabion	Desalted
PAEP-247_R	Pho		CCTTCCTGCTGCACACCTGCACCACGGNNNNCTGGAGCTGTCTGCGACTTT	Metabion	Desalted
IGFBP1-70_R	Pho		TGTTGCCACCTGTTAAAATGTACACTGNNNNCTGGAGCTGTCTGCGACTTT	Metabion	Desalted
DYNLT3-360_R	Pho		TTTGGAGAGGAATAAGCTTAGCGTTAANNNNCTGGAGCTGTCTGCGACTTT	Metabion	Desalted
CRABP2-160_R	Pho		CACTCCCCGCCTCCCCAAGTCAGCAGTNNNNCTGGAGCTGTCTGCGACTTT	Metabion	Desalted
NDRG1-1530_R	Pho		AGGTCTATTACGTGGGTGCCCTCTCCANNNNCTGGAGCTGTCTGCGACTTT	Metabion	Desalted
ID4-39_R	Pho		TGTCCAATCTCTTGTGATGTGTAACTTNNNNCTGGAGCTGTCTGCGACTTT	Metabion	Desalted
MMP7-44_R	Pho		TTGCAGTGGGTAGATGTCAATAAATGTNNNNCTGGAGCTGTCTGCGACTTT	Metabion	Desalted
ANXA4-1586_R	Pho		CTTGTTCTCTGTGGAGGAGATGATTAANNNNCTGGAGCTGTCTGCGACTTT	Metabion	Desalted
TSPAN8-99_R	Pho		GCTAGACCACAGATATCTTCTAGACATNNNNCTGGAGCTGTCTGCGACTTT	Metabion	Desalted
EDNRB-2862_R	Pho		TTGGTGAGCAAAAGATTCAAAAACTGCNNNNCTGGAGCTGTCTGCGACTTT	Metabion	Desalted
NNMT-833_R	Pho		AACGAAGGACTTTTCTCCCTGGTGGCGNNNNCTGGAGCTGTCTGCGACTTT	Metabion	Desalted
CLDN4-221_R	Pho		TCTGCCTCACCCGCTTCAGCCCAGGGCNNNNCTGGAGCTGTCTGCGACTTT	Metabion	Desalted
EFNA1-62_R	Pho		GTTGCTGTGTGTCTGTCCTGATTTCTANNNNCTGGAGCTGTCTGCGACTTT	Metabion	Desalted
COMP-138_R	Pho		CCATCAGCTGCGGCAAGCCTAGGGACCNNNNCTGGAGCTGTCTGCGACTTT	Metabion	Desalted
CD55-1548_R	Pho		ACCACTTCAGGTACTACCCGTCTTCTANNNNCTGGAGCTGTCTGCGACTTT	Metabion	Desalted
DKK1-175_R	Pho		CTACAAGAACGGAAGTGTGATATGTTTNNNNCTGGAGCTGTCTGCGACTTT	Metabion	Desalted
SPP1-313_R	Pho		AAAAGCTTCAGGGTTATGTCTATGTTCNNNNCTGGAGCTGTCTGCGACTTT	Metabion	Desalted
AQP3-170_R	Pho		GTCAGGTCATAAGTTTCATGTTTGCTTNNNNCTGGAGCTGTCTGCGACTTT	Metabion	Desalted

S100P-29_R	Pho		TTCCCCTAGGCTGAGCCTGCTCATGTANNNNCTGGAGCTGTCTGCGACTTT	Metabion	Desalted
APOD-94_R	Pho		ACCAATCAACCACGACAAAGGAAGTTGNNNNCTGGAGCTGTCTGCGACTTT	Metabion	Desalted
ACADSB-4320_R	Pho		CTTTTAAACTTGGGAAATAAGCACCTGNNNNCTGGAGCTGTCTGCGACTTT	Metabion	Desalted
C10orf10-368_R	Pho		GGAATCAGGCTGGGACTGATCAGAGGTNNNNCTGGAGCTGTCTGCGACTTT	Metabion	Desalted
ABCC3-4217_R	Pho		CAACGGAGCTGTGGCCGTGAAGATGCGNNNNCTGGAGCTGTCTGCGACTTT	Metabion	Desalted
TCN1-40_R	Pho		AACCTCCCCTTCTCTCTCTACATGTTCNNNNCTGGAGCTGTCTGCGACTTT	Metabion	Desalted
IDO1-153_R	Pho		GTAACAGAGCCACAAACTAATACTATGNNNNCTGGAGCTGTCTGCGACTTT	Metabion	Desalted
GPX3-34_R	Pho		TGGTCCACTGGCTTGAGACTCACCCCGNNNNCTGGAGCTGTCTGCGACTTT	Metabion	Desalted
BCL6-1192_R	Pho		AGTGTAACCTGCATTTCCGTCACAAAANNNNCTGGAGCTGTCTGCGACTTT	Metabion	Desalted
ANXA2-239_R	Pho		TGAAACACTTTGCCTCCTGTGTACTGTNNNNCTGGAGCTGTCTGCGACTTT	Metabion	Desalted
SFRP4-23_R	Pho		TAGGTGGTTTCTTCACTGACAATACTGNNNNCTGGAGCTGTCTGCGACTTT	Metabion	Desalted
SERPING1-81_R	Pho		TAGCCCTTCTCCATGGCCCTGCCATGCNNNNCTGGAGCTGTCTGCGACTTT	Metabion	Desalted
ARG2-34_R	Pho		ACCTCACAGAAATGTTAAACTGAGACANNNNCTGGAGCTGTCTGCGACTTT	Metabion	Desalted
C1R-156_R	Pho		GTAGTCCCATTGATGTACTTTACCTGANNNNCTGGAGCTGTCTGCGACTTT	Metabion	Desalted
C4BPA-161_R	Pho		TAGCAAATCTACTGTCAATTTGGCAGTNNNNCTGGAGCTGTCTGCGACTTT	Metabion	Desalted
GBP2-987_R	Pho		GCAACTTCAGAGTGTCAAACAACTGCCNNNNCTGGAGCTGTCTGCGACTTT	Metabion	Desalted
LAMB3-174_R	Pho		CAAAGATGGTGGAGATTGGCATGCCATNNNNCTGGAGCTGTCTGCGACTTT	Metabion	Desalted
ARID5B-3946_R	Pho		TCAAGCTGCCTTTCCATCTTCCCAGCTNNNNCTGGAGCTGTCTGCGACTTT	Metabion	Desalted
DPP4-427_R	Pho		GACTTCTAAGTAAAACCACAGCAGTTGNNNNCTGGAGCTGTCTGCGACTTT	Metabion	Desalted
SLC1A1-123_R	Pho		TTAGTGGCACTAGTTGGCAGAGCTGTTNNNNCTGGAGCTGTCTGCGACTTT	Metabion	Desalted
HABP2-18_R	Pho		TAGAGCTAGCTGACCTTTGGCCAAAAANNNNCTGGAGCTGTCTGCGACTTT	Metabion	Desalted
MAOA-59_R	Pho		GTGTGCTTTGCCACTGAAGTAAGATTTNNNNCTGGAGCTGTCTGCGACTTT	Metabion	Desalted
PRUNE2-190_R	Pho		GTTCCCTTAGAAACATTTAGATGTGCANNNNCTGGAGCTGTCTGCGACTTT	Metabion	Desalted
DDX52-5_R	Pho		CCTATCTGTTCTTTGTGTGTAAAGAGTNNNNCTGGAGCTGTCTGCGACTTT	Metabion	Desalted
CP-45_R	Pho		ATTGTCATGTCTCCAACAACAGTGAACNNNNCTGGAGCTGTCTGCGACTTT	Metabion	Desalted
MAP3K5-749_R	Pho		AAGGGGAGGGATGCTGTGCACACTGTGNNNNCTGGAGCTGTCTGCGACTTT	Metabion	Desalted
ENPEP-314_R	Pho		ACACATGTGCTGTAAATGAGAAATACCNNNNCTGGAGCTGTCTGCGACTTT	Metabion	Desalted
AOX1-528_R	Pho		TCAATCCATCCAGCTAAATGGAATAGGNNNNCTGGAGCTGTCTGCGACTTT	Metabion	Desalted
PPIA-1638_R	Pho		TCATTCCTTCTGTAGCTCAGGAGAGCANNNNCTGGAGCTGTCTGCGACTTT	Metabion	Desalted
CYC1-83_R	Pho		CTAAGCCTCTCTTCATCTGGAAGAAGANNNNCTGGAGCTGTCTGCGACTTT	Metabion	Desalted
YWHAZ-4145_R	Pho		TAACCGGCCTTCCAACTTTTGTCTGCCNNNNCTGGAGCTGTCTGCGACTTT	Metabion	Desalted
GAPDH-58_R	Pho		CCATGTAGACCCCTTGAAGAGGGGAGGNNNNCTGGAGCTGTCTGCGACTTT	Metabion	Desalted
HMBS-36_R	Pho		GAAGTCCAAGCAACAGCCTTTGAATGTNNNNCTGGAGCTGTCTGCGACTTT	Metabion	Desalted
TBP-343_R	Pho		TCTGGGCAGCGCTGCCCATTTATTTATNNNNCTGGAGCTGTCTGCGACTTT	Metabion	Desalted
SDHA-333_R	Pho		GGAATATAGACCCGTGATCGACAAAACNNNNCTGGAGCTGTCTGCGACTTT	Metabion	Desalted
ACTB-5_R	Pho		ACCAGTTGAATAAAAGTGCACACCTTANNNNCTGGAGCTGTCTGCGACTTT	Metabion	Desalted
chr2:101085535_L			ACACGACGCTCTTCCGATCTNNNNCGTTAGCGCCAATATAACGTCCGGGAT	Metabion	Desalted
chr2:11406302_L			ACACGACGCTCTTCCGATCTNNNNCTTTCTGTCTGGTTCTGTTGAACGCAA	Metabion	Desalted
chr2:120868127_L			ACACGACGCTCTTCCGATCTNNNNCTGAGAGCTCAGTCTTCGCCACTTCAA	Metabion	Desalted
chr2:135789621_L			ACACGACGCTCTTCCGATCTNNNNTTGCATCGGACCACAGAGGCGTAGAAC	Metabion	Desalted
chr2:163017782_L			ACACGACGCTCTTCCGATCTNNNNATCTGCAGGTTGCAAACACCGTGGGAT	Metabion	Desalted
chr2:178250714_L			ACACGACGCTCTTCCGATCTNNNNCTTCCACAAAGCAGAAGCCGTCTCATA	Metabion	Desalted
chr2:199463719_L			ACACGACGCTCTTCCGATCTNNNNACAAAAACGCCCTGGCGCGTGCAAAAT	Metabion	Desalted

chr2:21608302_L			ACACGACGCTCTTCCGATCTNNNNGCTGCATTTTCAGAGAAGGCCATCGTA	Metabion	Desalted
chr2:227759997_L			ACACGACGCTCTTCCGATCTNNNNCTGGGCTTCTTTCTAACCCCGCTGAAT	Metabion	Desalted
chr2:232372794_L			ACACGACGCTCTTCCGATCTNNNNGAATTGTGGACTGGACTGAGTACTCCT	Metabion	Desalted
chr2:23401307_L			ACACGACGCTCTTCCGATCTNNNNCCTCTCTCCATTCAGAAACCGTACTCT	Metabion	Desalted
chr2:237660339_L			ACACGACGCTCTTCCGATCTNNNNGGAGATGGCTTGGCAACTCACTGCGTA	Metabion	Desalted
chr2:240800370_L			ACACGACGCTCTTCCGATCTNNNNACCAATGCATTACTCAAGAGGCCCGAT	Metabion	Desalted
chr2:24739837_L			ACACGACGCTCTTCCGATCTNNNNTACGAAACTGTGTGAACGGTACCCGAA	Metabion	Desalted
chr2:3323538_L			ACACGACGCTCTTCCGATCTNNNNCCACGATGGACATGGGCCCTCAGCCAA	Metabion	Desalted
chr2:59344696_L			ACACGACGCTCTTCCGATCTNNNNTGCTTGAGTCCCAACTGGGGTGATAGC	Metabion	Desalted
chr2:71830719_L			ACACGACGCTCTTCCGATCTNNNNATCCCAGTCAGTTACAACGGCAGCAAT	Metabion	Desalted
chr2:82461489_L			ACACGACGCTCTTCCGATCTNNNNTATGATTCTCCTCTGACCCAGTCAACG	Metabion	Desalted
chr2:94878207_L			ACACGACGCTCTTCCGATCTNNNNTCCTGTCACCACGGAAGCCGCACTACT	Metabion	Desalted
chr2:98757977_L			ACACGACGCTCTTCCGATCTNNNNTGACAGTGCCAGGAACGCCCGTGAACT	Metabion	Desalted
chr2:100955305_L			ACACGACGCTCTTCCGATCTNNNNATGGAATGGGAAGGACAGCGACCCTTA	Metabion	Desalted
chr2:102305387_L			ACACGACGCTCTTCCGATCTNNNNGTGGAACAATCTGTAAGATCGGACGTT	Metabion	Desalted
chr2:103792960_L			ACACGACGCTCTTCCGATCTNNNNCTATATCAGATATTAACGGGCCCAGTT	Metabion	Desalted
chr2:104792858_L			ACACGACGCTCTTCCGATCTNNNNTCCATTTAAGATGGGAAACCGGAGTTG	Metabion	Desalted
chr2:108565153_L			ACACGACGCTCTTCCGATCTNNNNCAGGAGGTAACTTTTTCCTTAGTTGGA	Metabion	Desalted
chr2:112647051_L			ACACGACGCTCTTCCGATCTNNNNGTCTTACTGGGGGCCAAAGTGAGCGAA	Metabion	Desalted
chr2:118224692_L			ACACGACGCTCTTCCGATCTNNNNAGAAAGCCCGAAAAGGGAGGCGGTTAT	Metabion	Desalted
chr2:125311103_L			ACACGACGCTCTTCCGATCTNNNNTTTGGTTCTAAGTGCTACTCGCAAAGT	Metabion	Desalted
chr2:135073864_L			ACACGACGCTCTTCCGATCTNNNNCATCCCATGATGGGACCGTAAAAAACG	Metabion	Desalted
chr2:145245523_L			ACACGACGCTCTTCCGATCTNNNNTTGCCAACCCAAACAAAGTATAATCAG	Metabion	Desalted
chr2:152334950_L			ACACGACGCTCTTCCGATCTNNNNAAGTGACAATATGCTATCTCCAGAGAC	Metabion	Desalted
chr2:15474108_L			ACACGACGCTCTTCCGATCTNNNNTCACAAGGCGGTAGTTTTTAGTAATGG	Metabion	Desalted
chr2:167231349_L			ACACGACGCTCTTCCGATCTNNNNTTCCTACATGTCCTTGTAACGTCTCAT	Metabion	Desalted
chr2:17124032_L			ACACGACGCTCTTCCGATCTNNNNACAGGACAGCAAGAAACCCGTGGCAGT	Metabion	Desalted
chr2:176099389_L			ACACGACGCTCTTCCGATCTNNNNCTCCGCTGCAACTTAAAGCCGGTAGAA	Metabion	Desalted
chr2:177249947_L			ACACGACGCTCTTCCGATCTNNNNTCCTTAGGGGTGACTTTGACGGAACCA	Metabion	Desalted
chr2:17931415_L			ACACGACGCTCTTCCGATCTNNNNAAAGCCATGATGTGAGTACCGACTCCT	Metabion	Desalted
chr2:190246811_L			ACACGACGCTCTTCCGATCTNNNNCAAGGTCATTCTTCAAGTACGGGACAC	Metabion	Desalted
chr2:19240391_L			ACACGACGCTCTTCCGATCTNNNNTACTTTCAACATCAGAGCACGGGAGTC	Metabion	Desalted
chr2:197051977_L			ACACGACGCTCTTCCGATCTNNNNAATTATTCCCGTTTAAACTTCGGGGTT	Metabion	Desalted
chr2:198064874_L			ACACGACGCTCTTCCGATCTNNNNGCAAATAATTTCAGTGGACCGTTTGGT	Metabion	Desalted
chr2:202868115_L			ACACGACGCTCTTCCGATCTNNNNTATCACTGAATTGTACACTTCGAAACG	Metabion	Desalted
chr2:218819411_L			ACACGACGCTCTTCCGATCTNNNNAAGAAAGAACAACAGCGGATTAAGGAT	Metabion	Desalted
chr2:223957413_L			ACACGACGCTCTTCCGATCTNNNNGTTACGGGGACACTGGCCCGACTACTT	Metabion	Desalted
chr2:233308485_L			ACACGACGCTCTTCCGATCTNNNNCAGAGTATGCTTAATTCTAGACCGCTA	Metabion	Desalted
chr2:235577831_L			ACACGACGCTCTTCCGATCTNNNNAAGGCCCATGTCTGCTCGCTGGGACCT	Metabion	Desalted
chr2:2795811_L			ACACGACGCTCTTCCGATCTNNNNCCAGAAGACATTTAAAACTGATCAGTG	Metabion	Desalted
chr2:39665236_L			ACACGACGCTCTTCCGATCTNNNNCCAGATCAAGATTCCAAGGAGTTAAAC	Metabion	Desalted
chr2:42844903_L			ACACGACGCTCTTCCGATCTNNNNGGACCAGTTGTCCCATCGGGGCTTAGC	Metabion	Desalted
chr2:46512548_L			ACACGACGCTCTTCCGATCTNNNNTCCATAATCTTCAGTCGTTGGGTTTGC	Metabion	Desalted

chr2:47653669_L			ACACGACGCTCTTCCGATCTNNNNACTTATTGCTCACGATTGGCATACCAT	Metabion	Desalted
chr2:68077544_L			ACACGACGCTCTTCCGATCTNNNNCAGCCAAGATCAGCAGGTAGTACAACT	Metabion	Desalted
chr2:70411829_L			ACACGACGCTCTTCCGATCTNNNNCAAATGCCTGCTGCTAAGGATAGACGA	Metabion	Desalted
chr2:78404591_L			ACACGACGCTCTTCCGATCTNNNNTGAATGCCTGTCTGCAACGGCCTTGAT	Metabion	Desalted
chr2:88350766_L			ACACGACGCTCTTCCGATCTNNNNGCATTATGCAAAATAAAGCCGCCTTGT	Metabion	Desalted
chr2:96661714_L			ACACGACGCTCTTCCGATCTNNNNTCTTCAAGTCAGCGGTAGTCCCGATCA	Metabion	Desalted
chr2:98757977_L			ACACGACGCTCTTCCGATCTNNNNCTGACAGTGCCAGGAACGCCCGTGAAC	Metabion	Desalted
chr2:115162453_L			ACACGACGCTCTTCCGATCTNNNNCTGGCCAAAGCGACCCGAGCAGGCGAA	Metabion	Desalted
chr2:20646683_L			ACACGACGCTCTTCCGATCTNNNNGCCAGCCCTCTGCCAACGGCACCGAGT	Metabion	Desalted
chr2:238789650_L			ACACGACGCTCTTCCGATCTNNNNTAGTTCAGCGGGAGAAACCGATTCTAA	Metabion	Desalted
chr2:101085535_R	Pho		AACGATGCCCAAGCATGAGCAAGACAANNNNCTGGAGCTGTCTGCGACTTT	Metabion	Desalted
chr2:11406302_R	Pho		ACGACATCTGCTTCCCACTCCCTGAAANNNNCTGGAGCTGTCTGCGACTTT	Metabion	Desalted
chr2:120868127_R	Pho		TTCCCCGCGGTTTGAGCTGCAAGGAGGNNNNCTGGAGCTGTCTGCGACTTT	Metabion	Desalted
chr2:135789621_R	Pho		TTCGCTGATGCTTTGGGGATCCTTGGCNNNNCTGGAGCTGTCTGCGACTTT	Metabion	Desalted
chr2:163017782_R	Pho		AAGCCTCGTATCTGGGCCAACAGCAGANNNNCTGGAGCTGTCTGCGACTTT	Metabion	Desalted
chr2:178250714_R	Pho		TCGTGCACTATAAATGAGGACTTCCCTNNNNCTGGAGCTGTCTGCGACTTT	Metabion	Desalted
chr2:199463719_R	Pho		ACGAACGCCCACAGTTTGTCCCAACCCNNNNCTGGAGCTGTCTGCGACTTT	Metabion	Desalted
chr2:21608302_R	Pho		TTCCGTCCCCAATGGTTGTGGGCTTGTNNNNCTGGAGCTGTCTGCGACTTT	Metabion	Desalted
chr2:227759997_R	Pho		TCCTACACGGACCTCAGACGGATGCAGNNNNCTGGAGCTGTCTGCGACTTT	Metabion	Desalted
chr2:232372794_R	Pho		TACGTGGACCCTTTTAGGGACCACGAANNNNCTGGAGCTGTCTGCGACTTT	Metabion	Desalted
chr2:23401307_R	Pho		ATACACCCCCGGTCCCCACCCTAAGTCNNNNCTGGAGCTGTCTGCGACTTT	Metabion	Desalted
chr2:237660339_R	Pho		GTGTAGATATGGGGACATAGGGACCCCNNNNCTGGAGCTGTCTGCGACTTT	Metabion	Desalted
chr2:240800370_R	Pho		ACCACGCCGCACTGTGTGTGAAGGAATNNNNCTGGAGCTGTCTGCGACTTT	Metabion	Desalted
chr2:24739837_R	Pho		ACCTGATCAAAACCCAGTCACATTGCTNNNNCTGGAGCTGTCTGCGACTTT	Metabion	Desalted
chr2:3323538_R	Pho		CGTTCGCCGTGTTGTCAGCCTCCATGANNNNCTGGAGCTGTCTGCGACTTT	Metabion	Desalted
chr2:59344696_R	Pho		ATGCTACCGTCTTAATGTCCCCCCACCNNNNCTGGAGCTGTCTGCGACTTT	Metabion	Desalted
chr2:71830719_R	Pho		CGAAATCCAGCTTCTGATGTGAGATCTNNNNCTGGAGCTGTCTGCGACTTT	Metabion	Desalted
chr2:82461489_R	Pho		TTACACCGCTTTGCTTCTGGACCTATANNNNCTGGAGCTGTCTGCGACTTT	Metabion	Desalted
chr2:94878207_R	Pho		ACTTGTCGCAAACCACCAGTCACTACANNNNCTGGAGCTGTCTGCGACTTT	Metabion	Desalted
chr2:98757977_R	Pho		GCTTTCGTGGTTGCATGTGAAAACTTTNNNNCTGGAGCTGTCTGCGACTTT	Metabion	Desalted
chr2:100955305_R	Pho		CTGTACCCGCTTCCTGGGCCTAGCATGNNNNCTGGAGCTGTCTGCGACTTT	Metabion	Desalted
chr2:102305387_R	Pho		TCCTCTGATAACAGAAACTCCAGAGTTNNNNCTGGAGCTGTCTGCGACTTT	Metabion	Desalted
chr2:103792960_R	Pho		AACGACAGGCACACCTTAACTGCTAGANNNNCTGGAGCTGTCTGCGACTTT	Metabion	Desalted
chr2:104792858_R	Pho		CACATATCGCCTATGCCCACATTACAANNNNCTGGAGCTGTCTGCGACTTT	Metabion	Desalted
chr2:108565153_R	Pho		TACCGTCTTCGTTCCACAGAGTTTTTTNNNNCTGGAGCTGTCTGCGACTTT	Metabion	Desalted
chr2:112647051_R	Pho		ACCATCCGGAAGGGCTTGATTGACGTGNNNNCTGGAGCTGTCTGCGACTTT	Metabion	Desalted
chr2:118224692_R	Pho		TTACGACCGGCGGGTTGGAGTCTGGCANNNNCTGGAGCTGTCTGCGACTTT	Metabion	Desalted
chr2:125311103_R	Pho		CTTCACCGCATTTACACTGCTGGGATTNNNNCTGGAGCTGTCTGCGACTTT	Metabion	Desalted
chr2:135073864_R	Pho		TCTGTTGGGGACGCAAACTGCAGTTTCNNNNCTGGAGCTGTCTGCGACTTT	Metabion	Desalted
chr2:145245523_R	Pho		CGATGATCCGAGCAGACCAAGCTGTCTNNNNCTGGAGCTGTCTGCGACTTT	Metabion	Desalted
chr2:152334950_R	Pho		TACGGATCGCCTTTGCTGCAAATGGTCNNNNCTGGAGCTGTCTGCGACTTT	Metabion	Desalted
chr2:15474108_R	Pho		TTCGTGGGCGTTTCCGTGGTGGTGCAANNNNCTGGAGCTGTCTGCGACTTT	Metabion	Desalted
chr2:167231349_R	Pho		GGAACATACGTTACCAAAAAATAAGCCNNNNCTGGAGCTGTCTGCGACTTT	Metabion	Desalted

chr2:17124032_R	Pho		ATAGGCGAAATGGGTGTTTATTCTGGTNNNNCTGGAGCTGTCTGCGACTTT	Metabion	Desalted
chr2:176099389_R	Pho		GCAAGCCGGGCCCAGAAAGCCTGCGGANNNNCTGGAGCTGTCTGCGACTTT	Metabion	Desalted
chr2:177249947_R	Pho		AACTCTTCGGTTTTGCAAATTACCTCCNNNNCTGGAGCTGTCTGCGACTTT	Metabion	Desalted
chr2:17931415_R	Pho		CGTTTGAAGAGTCGTCTCTGTTTGAGANNNNCTGGAGCTGTCTGCGACTTT	Metabion	Desalted
chr2:190246811_R	Pho		GTATCTTAGCTGCAGGTGTGGCTGGATNNNNCTGGAGCTGTCTGCGACTTT	Metabion	Desalted
chr2:19240391_R	Pho		TGTACTCCGCTTTTGGCACTTCAGCAGNNNNCTGGAGCTGTCTGCGACTTT	Metabion	Desalted
chr2:197051977_R	Pho		ACCATCCGGGCAGTGCAGAGCTCTGACNNNNCTGGAGCTGTCTGCGACTTT	Metabion	Desalted
chr2:198064874_R	Pho		ATAGGGAAGCTTATGGAGACAGAAGCTNNNNCTGGAGCTGTCTGCGACTTT	Metabion	Desalted
chr2:202868115_R	Pho		GTTGTTTGGGTTTGTTTAGCCAAAGTANNNNCTGGAGCTGTCTGCGACTTT	Metabion	Desalted
chr2:218819411_R	Pho		TGTTGGTTCCGCACAGCAGATAGACATNNNNCTGGAGCTGTCTGCGACTTT	Metabion	Desalted
chr2:223957413_R	Pho		TCGTTCCGTCTTCCATCGTTTTCTCTCNNNNCTGGAGCTGTCTGCGACTTT	Metabion	Desalted
chr2:233308485_R	Pho		AGATGGCCTCTTAATTGTAAAACAGAGNNNNCTGGAGCTGTCTGCGACTTT	Metabion	Desalted
chr2:235577831_R	Pho		GATAGTTCGCCTTTGTACGGATGAAAANNNNCTGGAGCTGTCTGCGACTTT	Metabion	Desalted
chr2:2795811_R	Pho		TACGCGCGAGAGTAGCCAAAGACTCAGNNNNCTGGAGCTGTCTGCGACTTT	Metabion	Desalted
chr2:39665236_R	Pho		GCTACCACGCAGCCCGACCTGGATGGGNNNNCTGGAGCTGTCTGCGACTTT	Metabion	Desalted
chr2:42844903_R	Pho		ATCGACTCTCTTTCTCTGGCAGATGTTNNNNCTGGAGCTGTCTGCGACTTT	Metabion	Desalted
chr2:46512548_R	Pho		ACGAGGCGTCCTTTCTCAATGTTAAACNNNNCTGGAGCTGTCTGCGACTTT	Metabion	Desalted
chr2:47653669_R	Pho		TAGGGGATTGTTGGCCATCCCCTCTTCNNNNCTGGAGCTGTCTGCGACTTT	Metabion	Desalted
chr2:68077544_R	Pho		TAGCCGAACCCTTCAGGCTCCAGAGAANNNNCTGGAGCTGTCTGCGACTTT	Metabion	Desalted
chr2:70411829_R	Pho		CTCACCCGGTCCTTGGGAATGCTTATANNNNCTGGAGCTGTCTGCGACTTT	Metabion	Desalted
chr2:78404591_R	Pho		CATACTACCAGGGTTTCGACAGCCTTTNNNNCTGGAGCTGTCTGCGACTTT	Metabion	Desalted
chr2:88350766_R	Pho		TTCCCGCGCCTGGTAACCCCGGGCTTCNNNNCTGGAGCTGTCTGCGACTTT	Metabion	Desalted
chr2:96661714_R	Pho		ACATGGCTTACTTACAGCTCTTCATCTNNNNCTGGAGCTGTCTGCGACTTT	Metabion	Desalted
chr2:98757977_R	Pho		TGCTTTCGTGGTTGCATGTGAAAACTTNNNNCTGGAGCTGTCTGCGACTTT	Metabion	Desalted
chr2:115162453_R	Pho		TGACCTTTAGGCGGACGGGGTTTTCCCNNNNCTGGAGCTGTCTGCGACTTT	Metabion	Desalted
chr2:20646683_R	Pho		TCACGCAGTGTGCACGCGCGGCCTGGTNNNNCTGGAGCTGTCTGCGACTTT	Metabion	Desalted
chr2:238789650_R	Pho		GGTTGCCGTATAATTAGCAGGGTCTCCNNNNCTGGAGCTGTCTGCGACTTT	Metabion	Desalted
chr21:14788887_L			ACACGACGCTCTTCCGATCTNNNNAACCACTTTCCTTCTGTGGTAGCCGAT	Metabion	Desalted
chr21:15967507_L			ACACGACGCTCTTCCGATCTNNNNCAACCTGAGGCTTGTGATCGGCATGAA	Metabion	Desalted
chr21:20262845_L			ACACGACGCTCTTCCGATCTNNNNGATACATCTGAGAGATACGCGGAGATA	Metabion	Desalted
chr21:25447962_L			ACACGACGCTCTTCCGATCTNNNNCAAAACTCAATGCCAAGTGGTTGAACG	Metabion	Desalted
chr21:27008952_L			ACACGACGCTCTTCCGATCTNNNNTCATACTTGTCTCCCCAGTCCCGCTCA	Metabion	Desalted
chr21:29364391_L			ACACGACGCTCTTCCGATCTNNNNCATGAGAGAAGGGCCAGTACCTTTTGC	Metabion	Desalted
chr21:31658073_L			ACACGACGCTCTTCCGATCTNNNNTTGTTCAGGTGTGACGACCATCCTACG	Metabion	Desalted
chr21:32848925_L			ACACGACGCTCTTCCGATCTNNNNTAATGCTGCAAATACCCGTGCAAGACT	Metabion	Desalted
chr21:33550726_L			ACACGACGCTCTTCCGATCTNNNNGTAGCAATGGAGTTGACCGAACAACCT	Metabion	Desalted
chr21:34761453_L			ACACGACGCTCTTCCGATCTNNNNTGGTCCTGGTCCTCAGTGGAACCCGTT	Metabion	Desalted
chr21:36460959_L			ACACGACGCTCTTCCGATCTNNNNAAGCAGGCTGTGGGGACTCACACGTAG	Metabion	Desalted
chr21:39062244_L			ACACGACGCTCTTCCGATCTNNNNCATGAGTCCCCATGCCTGATCCCAGAC	Metabion	Desalted
chr21:40995931_L			ACACGACGCTCTTCCGATCTNNNNCCTCCTCTAGCTGATGCTGTGGCAGTC	Metabion	Desalted
chr21:41943683_L			ACACGACGCTCTTCCGATCTNNNNTGCAAGTGAGCAAGCGATGAGGTTACG	Metabion	Desalted
chr21:42360636_L			ACACGACGCTCTTCCGATCTNNNNCCCCTATGTCTCATGGGCCACATAGA	Metabion	Desalted
chr21:43812691_L			ACACGACGCTCTTCCGATCTNNNNCCACTGCATGTCAGCGCCCAGCCGTAG	Metabion	Desalted

chr21:44527351_L			ACACGACGCTCTTCCGATCTNNNNATGAGACGAACTTCTCTTCGGTCCACT	Metabion	Desalted
chr21:44855283_L			ACACGACGCTCTTCCGATCTNNNNCCAAACGACAGCGCACGGTGGTGTAAC	Metabion	Desalted
chr21:45828339_L			ACACGACGCTCTTCCGATCTNNNNGCCCAGACTCTTAATACGGTGAGTTAC	Metabion	Desalted
chr21:46415136_L			ACACGACGCTCTTCCGATCTNNNNGTTCCTGGGTCCACACTGCGTGCACCT	Metabion	Desalted
chr21:14853307_L			ACACGACGCTCTTCCGATCTNNNNGAATGAGTTCTCACTCTACGAGTTCAC	Metabion	Desalted
chr21:15494467_L			ACACGACGCTCTTCCGATCTNNNNCCCTCCAGCTTACCGTGGGTATTCAAC	Metabion	Desalted
chr21:16486166_L			ACACGACGCTCTTCCGATCTNNNNAGATGTGAAGACAGCACACCGCTAGGT	Metabion	Desalted
chr21:18477249_L			ACACGACGCTCTTCCGATCTNNNNTGTTTGAGAATTACTGCGTTACACCAA	Metabion	Desalted
chr21:18745264_L			ACACGACGCTCTTCCGATCTNNNNTCTTCAATTCACAAACTAACGCAGTCA	Metabion	Desalted
chr21:23049826_L			ACACGACGCTCTTCCGATCTNNNNATCTCCTTGCATGATCCAAGCACCGTT	Metabion	Desalted
chr21:24075508_L			ACACGACGCTCTTCCGATCTNNNNAAATCTAAAGATCTCTGCCTTCGCTCT	Metabion	Desalted
chr21:24844831_L			ACACGACGCTCTTCCGATCTNNNNATCAGTAGGATAAACAACCGACGTTCT	Metabion	Desalted
chr21:25930186_L			ACACGACGCTCTTCCGATCTNNNNCATTTTGTAGTTTCAGTGAGTCGTGTC	Metabion	Desalted
chr21:26381471_L			ACACGACGCTCTTCCGATCTNNNNTAAGTGAACCACTGACATATTGGAGTT	Metabion	Desalted
chr21:26966048_L			ACACGACGCTCTTCCGATCTNNNNGGCACGAAGCCAGCAATGCCCACCGAA	Metabion	Desalted
chr21:29073899_L			ACACGACGCTCTTCCGATCTNNNNACCGTAGTCAGTAGTCACGGCGTTAGA	Metabion	Desalted
chr21:31120259_L			ACACGACGCTCTTCCGATCTNNNNTGTGCAAGAGCGCGACCTAAGGGGACA	Metabion	Desalted
chr21:31956365_L			ACACGACGCTCTTCCGATCTNNNNGTGCCAGAAGGTTTCCATCCATAAAAG	Metabion	Desalted
chr21:32338779_L			ACACGACGCTCTTCCGATCTNNNNGCATCACGTAGACCACCGGGAGCTGGA	Metabion	Desalted
chr21:33024001_L			ACACGACGCTCTTCCGATCTNNNNAATTGAACGGTTATGGGTCATCCTTGT	Metabion	Desalted
chr21:33127293_L			ACACGACGCTCTTCCGATCTNNNNAGAAAAGACTGCCGTGGGGATCGGTTT	Metabion	Desalted
chr21:33915819_L			ACACGACGCTCTTCCGATCTNNNNGCGCGCGTTGGCGTAACCGCTAGGTTC	Metabion	Desalted
chr21:34074498_L			ACACGACGCTCTTCCGATCTNNNNGGATGCTAAGCGAACCAGCGGCCCCTT	Metabion	Desalted
chr21:35048563_L			ACACGACGCTCTTCCGATCTNNNNAGCACAACTTACTCGCACTTGACAAAG	Metabion	Desalted
chr21:35205270_L			ACACGACGCTCTTCCGATCTNNNNCTAGCAGTTAGACGGTCCATCTTTCTC	Metabion	Desalted
chr21:36708786_L			ACACGACGCTCTTCCGATCTNNNNCAAATCGATATCCCCGTTTGGCCACGA	Metabion	Desalted
chr21:36881369_L			ACACGACGCTCTTCCGATCTNNNNTGTCTAACAGGGGCATGGAACTCATTC	Metabion	Desalted
chr21:38759623_L			ACACGACGCTCTTCCGATCTNNNNATTTTCACTTAAACACAGCCCTGTCTG	Metabion	Desalted
chr21:39444854_L			ACACGACGCTCTTCCGATCTNNNNTTGGCTTGGGGAATTATTGAGCGCTAT	Metabion	Desalted
chr21:40178954_L			ACACGACGCTCTTCCGATCTNNNNCGTTGCTGGGCTCGCTCTTGCCAATCC	Metabion	Desalted
chr21:41506859_L			ACACGACGCTCTTCCGATCTNNNNGGCATTGCCTTGGGCGCGATGCGCTCA	Metabion	Desalted
chr21:41768876_L			ACACGACGCTCTTCCGATCTNNNNCCTCTGCGGACTTTGAAGTGCTTTACC	Metabion	Desalted
chr21:42138015_L			ACACGACGCTCTTCCGATCTNNNNACACACCCACTGGACTGGCTCCACGAT	Metabion	Desalted
chr21:42254610_L			ACACGACGCTCTTCCGATCTNNNNCGTGGCTTTGCCACATGATCACGAAAA	Metabion	Desalted
chr21:43273281_L			ACACGACGCTCTTCCGATCTNNNNGCCTTTATCAGGGCGTGAATCCCACGA	Metabion	Desalted
chr21:43319469_L			ACACGACGCTCTTCCGATCTNNNNACATCTGCTCCGGGCGATGTGACTCAG	Metabion	Desalted
chr21:44326763_L			ACACGACGCTCTTCCGATCTNNNNGAAGATGCTGGCACAATACCGCATCAG	Metabion	Desalted
chr21:44708346_L			ACACGACGCTCTTCCGATCTNNNNGCACACAGCCTTCCAGGAGCGGACTTG	Metabion	Desalted
chr21:44771421_L			ACACGACGCTCTTCCGATCTNNNNGAACTGCTCCCGGTCATCGCGCCACAT	Metabion	Desalted
chr21:45056373_L			ACACGACGCTCTTCCGATCTNNNNCTTCCTGCACCTTCCAGCGTCTTGTAT	Metabion	Desalted
chr21:45254594_L			ACACGACGCTCTTCCGATCTNNNNTTTCTCTTTGGCCGCGTTGCGGGAAAA	Metabion	Desalted
chr21:45982832_L			ACACGACGCTCTTCCGATCTNNNNGGAACACGGGTGCGACGGCCTCAACCT	Metabion	Desalted
chr21:46125933_L			ACACGACGCTCTTCCGATCTNNNNCCTCAAGTTTGCCTACGACCGCCTCAT	Metabion	Desalted

chr21:46370078_L			ACACGACGCTCTTCCGATCTNNNNCTTCATCAGAGCGTTCAGGCACTTACG	Metabion	Desalted
chr21:14788887_R	Pho		TTCCCACTGATTCCTGTCTCCTGCTATNNNNCTGGAGCTGTCTGCGACTTT	Metabion	Desalted
chr21:15967507_R	Pho		AGGTAGCGGCAGTCTTATGGGACTGAGNNNNCTGGAGCTGTCTGCGACTTT	Metabion	Desalted
chr21:20262845_R	Pho		CATCTCCGAGTTTGAAATCACCACACANNNNCTGGAGCTGTCTGCGACTTT	Metabion	Desalted
chr21:25447962_R	Pho		CTTAGCCACAGAACATACTGAGACTCTNNNNCTGGAGCTGTCTGCGACTTT	Metabion	Desalted
chr21:27008952_R	Pho		TGTCCCTGTATACCAAATGGCCAGAACNNNNCTGGAGCTGTCTGCGACTTT	Metabion	Desalted
chr21:29364391_R	Pho		GAAGTCACTAGGTGGACCTTGAGGAATNNNNCTGGAGCTGTCTGCGACTTT	Metabion	Desalted
chr21:31658073_R	Pho		AAGGCACCACCCAGGCATCATTAGACCNNNNCTGGAGCTGTCTGCGACTTT	Metabion	Desalted
chr21:32848925_R	Pho		TAGACGCTGATAAGAGAGGAGGTGGTANNNNCTGGAGCTGTCTGCGACTTT	Metabion	Desalted
chr21:33550726_R	Pho		GTGACGACGACAGAGTTGGAGCAGCCTNNNNCTGGAGCTGTCTGCGACTTT	Metabion	Desalted
chr21:34761453_R	Pho		TTGTTTCGATTGTCCCTGACCTGGCCTNNNNCTGGAGCTGTCTGCGACTTT	Metabion	Desalted
chr21:36460959_R	Pho		TCGTTCAGCCTGTACCCGCTGTGCGTGNNNNCTGGAGCTGTCTGCGACTTT	Metabion	Desalted
chr21:39062244_R	Pho		GTTCTACCCACAGCTGCCCACGGCAGGNNNNCTGGAGCTGTCTGCGACTTT	Metabion	Desalted
chr21:40995931_R	Pho		TAGCGGACAAGAGCAACATCATCACAANNNNCTGGAGCTGTCTGCGACTTT	Metabion	Desalted
chr21:41943683_R	Pho		CTGTGGCTATTTCTCAAGAATGCCCAANNNNCTGGAGCTGTCTGCGACTTT	Metabion	Desalted
chr21:42360636_R	Pho		ATTGCACGGCCACTTCTGGCTAAAAGANNNNCTGGAGCTGTCTGCGACTTT	Metabion	Desalted
chr21:43812691_R	Pho		ACGAGGGTTTGGGAGGCATGGCTGGGCNNNNCTGGAGCTGTCTGCGACTTT	Metabion	Desalted
chr21:44527351_R	Pho		TGTAGACGGCGGATGTGGCTTTGCGATNNNNCTGGAGCTGTCTGCGACTTT	Metabion	Desalted
chr21:44855283_R	Pho		TGCTAGACACGCCCTTCCGTGTCCCTCNNNNCTGGAGCTGTCTGCGACTTT	Metabion	Desalted
chr21:45828339_R	Pho		TCTCGCTCTGGATCTGCCCCCTCGTGCNNNNCTGGAGCTGTCTGCGACTTT	Metabion	Desalted
chr21:46415136_R	Pho		CATTGGCGTTTAGAGCCTGAAAGATTCNNNNCTGGAGCTGTCTGCGACTTT	Metabion	Desalted
chr21:14853307_R	Pho		GTGATGTACTGGCTCCCTCTTTGCCTTNNNNCTGGAGCTGTCTGCGACTTT	Metabion	Desalted
chr21:15494467_R	Pho		TTGTGCCGGCGTCGATACTTCCACAGGNNNNCTGGAGCTGTCTGCGACTTT	Metabion	Desalted
chr21:16486166_R	Pho		CTTGCACGGGCCTGCTGTAACCATTCCNNNNCTGGAGCTGTCTGCGACTTT	Metabion	Desalted
chr21:18477249_R	Pho		TTGTGACTCACTTCCAGCGGCTGGATANNNNCTGGAGCTGTCTGCGACTTT	Metabion	Desalted
chr21:18745264_R	Pho		GTGACTCGATTTCAGCCGGTTGCAGAANNNNCTGGAGCTGTCTGCGACTTT	Metabion	Desalted
chr21:23049826_R	Pho		ACTAGGCTCCACCTCCAACACCGGGGANNNNCTGGAGCTGTCTGCGACTTT	Metabion	Desalted
chr21:24075508_R	Pho		CCTATTGGGACTTGTTATAAGGCGATCNNNNCTGGAGCTGTCTGCGACTTT	Metabion	Desalted
chr21:24844831_R	Pho		TCAGCGTACCGTGTTGTCAGATGATTCNNNNCTGGAGCTGTCTGCGACTTT	Metabion	Desalted
chr21:25930186_R	Pho		CCTAAAGCCGTGTCCTGTGTGACCAGCNNNNCTGGAGCTGTCTGCGACTTT	Metabion	Desalted
chr21:26381471_R	Pho		ACGACAGTTGCCTGTTTTGACCTTGACNNNNCTGGAGCTGTCTGCGACTTT	Metabion	Desalted
chr21:26966048_R	Pho		CCATCTCGCTCCAGGTCCAAGAGGAACNNNNCTGGAGCTGTCTGCGACTTT	Metabion	Desalted
chr21:29073899_R	Pho		TTTTTGCGGTTTTTGATCTGGAAGCCANNNNCTGGAGCTGTCTGCGACTTT	Metabion	Desalted
chr21:31120259_R	Pho		TTCTTGTCGACGGTACAGGAGGGTGGGNNNNCTGGAGCTGTCTGCGACTTT	Metabion	Desalted
chr21:31956365_R	Pho		CGATGTGTCCAAGTCCTCTTTGTATGCNNNNCTGGAGCTGTCTGCGACTTT	Metabion	Desalted
chr21:32338779_R	Pho		TGAGCTTCGTGCGCACGGAGCTTTCTGNNNNCTGGAGCTGTCTGCGACTTT	Metabion	Desalted
chr21:33024001_R	Pho		AACCGTTGGACGACATAACACCACGCTNNNNCTGGAGCTGTCTGCGACTTT	Metabion	Desalted
chr21:33127293_R	Pho		CTGTTCCGAGAGTACATAGCAGAGTGANNNNCTGGAGCTGTCTGCGACTTT	Metabion	Desalted
chr21:33915819_R	Pho		TCTGGGAAGTGTAGGCGTAGGGCGTCANNNNCTGGAGCTGTCTGCGACTTT	Metabion	Desalted
chr21:34074498_R	Pho		TCAGGTGACGGCGTGGCCAAGGACAGGNNNNCTGGAGCTGTCTGCGACTTT	Metabion	Desalted
chr21:35048563_R	Pho		TTCTCACGCACCGACTGAACACTCCAANNNNCTGGAGCTGTCTGCGACTTT	Metabion	Desalted
chr21:35205270_R	Pho		TATCAGCCGTTTAGCAGCCTCTACTTTNNNNCTGGAGCTGTCTGCGACTTT	Metabion	Desalted
chr21:36708786_R	Pho		GAATGGCGATTTCAAAGCAGATTAGATNNNNCTGGAGCTGTCTGCGACTTT	Metabion	Desalted

chr21:36881369_R	Pho		GACTTTCCCTGGGTTCCAGAAGGAAACNNNNCTGGAGCTGTCTGCGACTTT	Metabion	Desalted
chr21:38759623_R	Pho		CGATGCCAACAGACTTTAGCTCAATTTNNNNCTGGAGCTGTCTGCGACTTT	Metabion	Desalted
chr21:39444854_R	Pho		CTTGGACGAGCTGTGTTTGAGATGCCGNNNNCTGGAGCTGTCTGCGACTTT	Metabion	Desalted
chr21:40178954_R	Pho		GGTTCTTGGCGTACATGCGGATGCTGTNNNNCTGGAGCTGTCTGCGACTTT	Metabion	Desalted
chr21:41506859_R	Pho		TCTTTCCCGCGGGACCACTGCACAGGTNNNNCTGGAGCTGTCTGCGACTTT	Metabion	Desalted
chr21:41768876_R	Pho		GATTCACATGACAACTGGTAAAACGAANNNNCTGGAGCTGTCTGCGACTTT	Metabion	Desalted
chr21:42138015_R	Pho		ACCAGCGGCAGTGCTATATGGGTGACCNNNNCTGGAGCTGTCTGCGACTTT	Metabion	Desalted
chr21:42254610_R	Pho		TGGAGGCGGTCGATGAGAAGGCCCTTANNNNCTGGAGCTGTCTGCGACTTT	Metabion	Desalted
chr21:43273281_R	Pho		ACACGGCTCCACCCTGAGGATCTCCCCNNNNCTGGAGCTGTCTGCGACTTT	Metabion	Desalted
chr21:43319469_R	Pho		CTGTGGACGATGACGACATGATCCTGANNNNCTGGAGCTGTCTGCGACTTT	Metabion	Desalted
chr21:44326763_R	Pho		TATGGCCGCCTACGTGTCAGGGGAGCTNNNNCTGGAGCTGTCTGCGACTTT	Metabion	Desalted
chr21:44708346_R	Pho		GAGACCTCGCCAAGGACCAGGACTCCCNNNNCTGGAGCTGTCTGCGACTTT	Metabion	Desalted
chr21:44771421_R	Pho		TTTGGACGCATCCACGTTAGCTCCACTNNNNCTGGAGCTGTCTGCGACTTT	Metabion	Desalted
chr21:45056373_R	Pho		TCGTGGAAGGAGAGAATGAGCTGGAACNNNNCTGGAGCTGTCTGCGACTTT	Metabion	Desalted
chr21:45254594_R	Pho		ATTTCTGCTGCTCACGAGTAGAAACACNNNNCTGGAGCTGTCTGCGACTTT	Metabion	Desalted
chr21:45982832_R	Pho		CCTAAGGTTGGGCGAGCGTTGCCCTGANNNNCTGGAGCTGTCTGCGACTTT	Metabion	Desalted
chr21:46125933_R	Pho		CAAGGAGAGCCGGCGCCAGAAGACACGNNNNCTGGAGCTGTCTGCGACTTT	Metabion	Desalted
chr21:46370078_R	Pho		GATACACTTGGAGCCGCTGGATTGTGCNNNNCTGGAGCTGTCTGCGACTTT	Metabion	Desalted
microRNA universal	Pho		AGATCGGAAGAGCACACGTCTCTGGAGCTGTCTGCGACTTT	Metabion	Desalted
hsa-miR-21-5p			ACGACGCTCTTCCGATCTNNNNNNNNTAGCTTATCAGACTGATGTTGA	Metabion	Desalted
hsa-miR-449a			ACGACGCTCTTCCGATCTNNNNNNNNTGGCAGTGTATTGTTAGCTGGT	Metabion	Desalted
hsa-miR-151a-5p			ACGACGCTCTTCCGATCTNNNNNNNNTCGAGGAGCTCACAGTCTAGT	Metabion	Desalted
hsa-miR-196b-5p			ACGACGCTCTTCCGATCTNNNNNNNNTAGGTAGTTTCCTGTTGTTGGG	Metabion	Desalted
hsa-miR-191-5p			ACGACGCTCTTCCGATCTNNNNNNNNcaacggaatcccaaaagcagctg	Metabion	Desalted
hsa-miR-127-3p			ACGACGCTCTTCCGATCTNNNNNNNNtcggatccgtctgagcttggct	Metabion	Desalted
hsa-miR-186-5p			ACGACGCTCTTCCGATCTNNNNNNNNcaaagaattctccttttgggct	Metabion	Desalted
hsa-miR-182-5p			ACGACGCTCTTCCGATCTNNNNNNNNtttggcaatggtagaactcacact	Metabion	Desalted
hsa-miR-21-3p			ACGACGCTCTTCCGATCTNNNNNNNNcaacaccagtcgatgggctgt	Metabion	Desalted
hsa-miR-126-3p			ACGACGCTCTTCCGATCTNNNNNNNNtcgtaccgtgagtaataatgcg	Metabion	Desalted
hsa-miR-30b-5p			ACGACGCTCTTCCGATCTNNNNNNNNtgtaaacatcctacactcagct	Metabion	Desalted
hsa-miR-221-3p			ACGACGCTCTTCCGATCTNNNNNNNNagctacattgtctgctgggtttc	Metabion	Desalted
hsa-miR-411-5p			ACGACGCTCTTCCGATCTNNNNNNNNtagtagaccgtatagcgtacg	Metabion	Desalted
hsa-miR-429			ACGACGCTCTTCCGATCTNNNNNNNNtaatactgtctggtaaaaccgt	Metabion	Desalted
hsa-miR-93-5p			ACGACGCTCTTCCGATCTNNNNNNNNcaaagtgctgttcgtgcaggtag	Metabion	Desalted
hsa-miR-24-3p			ACGACGCTCTTCCGATCTNNNNNNNNtggctcagttcagcaggaacag	Metabion	Desalted
hsa-miR-532-5p			ACGACGCTCTTCCGATCTNNNNNNNNcatgccttgagtgtaggaccgt	Metabion	Desalted
hsa-miR-345-5p			ACGACGCTCTTCCGATCTNNNNNNNNgctgactcctagtccagggctc	Metabion	Desalted
hsa-miR-140-3p			ACGACGCTCTTCCGATCTNNNNNNNNtaccacagggtagaaccacgg	Metabion	Desalted
hsa-miR-31-5p			ACGACGCTCTTCCGATCTNNNNNNNNaggcaagatgctggcatagct	Metabion	Desalted
hsa-miR-136-3p			ACGACGCTCTTCCGATCTNNNNNNNNcatcatcgtctcaaatgagtct	Metabion	Desalted
hsa-miR-28-5p			ACGACGCTCTTCCGATCTNNNNNNNNaaggagctcacagtctattgag	Metabion	Desalted
hsa-miR-484			ACGACGCTCTTCCGATCTNNNNNNNNtcaggctcagtcccctcccgat	Metabion	Desalted
hsa-miR-210-3p			ACGACGCTCTTCCGATCTNNNNNNNNctgtgcgtgtgacagcggctga	Metabion	Desalted

hsa-miR-128-3p			ACGACGCTCTTCCGATCTNNNNNNNNtcacagtgaaccggtctcttt	Metabion	Desalted
hsa-miR-363-3p			ACGACGCTCTTCCGATCTNNNNNNNNaattgcacggtatccatctgta	Metabion	Desalted
hsa-miR-183-5p			ACGACGCTCTTCCGATCTNNNNNNNNtatggcactggtagaattcact	Metabion	Desalted
hsa-miR-542-3p			ACGACGCTCTTCCGATCTNNNNNNNNtgtgacagattgataactgaaa	Metabion	Desalted
hsa-miR-335-5p			ACGACGCTCTTCCGATCTNNNNNNNNtcaagagcaataacgaaaaatgt	Metabion	Desalted
hsa-miR-342-3p			ACGACGCTCTTCCGATCTNNNNNNNNtctcacacagaaatcgcacccgt	Metabion	Desalted
hsa-miR-425-5p			ACGACGCTCTTCCGATCTNNNNNNNNaatgacacgatcactcccgttga	Metabion	Desalted
hsa-miR-421			ACGACGCTCTTCCGATCTNNNNNNNNatcaacagacattaattgggcgc	Metabion	Desalted
hsa-miR-454-3p			ACGACGCTCTTCCGATCTNNNNNNNNtagtgcaatattgcttatagggt	Metabion	Desalted
hsa-miR-361-5p			ACGACGCTCTTCCGATCTNNNNNNNNttatcagaatctccaggggtac	Metabion	Desalted
hsa-miR-214-5p			ACGACGCTCTTCCGATCTNNNNNNNNtgcctgtctacacttgctgtgc	Metabion	Desalted
hsa-miR-221-5p			ACGACGCTCTTCCGATCTNNNNNNNNacctggcatacaatgtagattt	Metabion	Desalted
hsa-miR-136-5p			ACGACGCTCTTCCGATCTNNNNNNNNactccatttgttttgatgatgga	Metabion	Desalted
hsa-miR-493-5p			ACGACGCTCTTCCGATCTNNNNNNNNttgtacatggtaggctttcatt	Metabion	Desalted
hsa-miR-17-3p			ACGACGCTCTTCCGATCTNNNNNNNNactgcagtgaaggcacttgtag	Metabion	Desalted
hsa-miR-493-3p			ACGACGCTCTTCCGATCTNNNNNNNNtgaaggtctactgtgtgccagg	Metabion	Desalted
hsa-miR-140-5p			ACGACGCTCTTCCGATCTNNNNNNNNcagtggttttaccctatggtag	Metabion	Desalted
hsa-miR-143-5p			ACGACGCTCTTCCGATCTNNNNNNNNggtgcagtgctgcatctctggt	Metabion	Desalted
hsa-miR-330-5p			ACGACGCTCTTCCGATCTNNNNNNNNtctctgggcctgtgtcttaggc	Metabion	Desalted
hsa-miR-431-5p			ACGACGCTCTTCCGATCTNNNNNNNNtgtcttgcaggccgtcatgca	Metabion	Desalted
hsa-miR-542-5p			ACGACGCTCTTCCGATCTNNNNNNNNtcggggatcatcatgtcacgaga	Metabion	Desalted
hsa-miR-432-5p			ACGACGCTCTTCCGATCTNNNNNNNNtcttggagtaggtcattgggtgg	Metabion	Desalted
hsa-miR-505-3p			ACGACGCTCTTCCGATCTNNNNNNNNcgtcaacacttgctggtttcct	Metabion	Desalted
hsa-miR-324-3p			ACGACGCTCTTCCGATCTNNNNNNNNactgccccaggtgctgctgg	Metabion	Desalted
hsa-miR-377-3p			ACGACGCTCTTCCGATCTNNNNNNNNatcacacaaaggcaacttttgt	Metabion	Desalted
hsa-miR-454-5p			ACGACGCTCTTCCGATCTNNNNNNNNaccctatcaatattgtctctgc	Metabion	Desalted

NNNN are four random nucleotides used as a unique molecular identifier (UMI)

NNNNNNNN are eight random nucleotides used as a unique molecular identifier (UMI)

LNA bases are +G
